# Risk factors for mortality in anti-MDA5 antibody-positive dermatomyositis with interstitial lung disease: a systematic review and meta-analysis

**DOI:** 10.3389/fimmu.2025.1628748

**Published:** 2025-07-17

**Authors:** Yahui Yang, Ying Li, Weiwei Yuan, Shijie Zhang, Xing He, Jiaqi Ji

**Affiliations:** ^1^ School of Medicine, University of Electronic Science and Technology of China, Chengdu, China; ^2^ Department of Otorhinolaryngology, The First People’s Hospital of Shuangliu District/West China (Airport) Hospital Sichuan University, Chengdu, China; ^3^ Department of Pulmonary and Critical Care Medicine, West China Hospital, Sichuan University, Chengdu, China; ^4^ State Key Laboratory of Respiratory Health and Multimorbidity, West China Hospital, Sichuan University, Chengdu, China; ^5^ Department of Pulmonary and Critical Care Medicine, Sichuan Provincial People’s Hospital, School of Medicine, University of Electronic Science and Technology of China, Chengdu, China

**Keywords:** anti-melanoma differentiation-associated protein 5, dermatomyositis, interstitial lung disease, mortality, poor prognosis

## Abstract

**Background:**

Anti-melanoma differentiation-associated gene 5 (MDA5) antibody-positive dermatomyositis with interstitial lung disease (MDA5^+^ DM-ILD) carries a high mortality risk. This meta-analysis aimed to identify mortality risk factors to guide early clinical intervention.

**Methods:**

Following PRISMA guidelines, we systematically searched PubMed, Embase, Web of Science, and Scopus for studies published before November 18, 2024. Pooled hazard ratios (HRs) with 95% confidence intervals (CIs) were calculated for mortality risk factors. Heterogeneity, sensitivity, and publication bias were assessed using Cochran’s Q, one-by-one elimination, and Egger’s tests, respectively.

**Results:**

Among 1,153 patients from 15 studies, significant risk factors for mortality included older age (HR = 1.04, 95%CI: 1.03, 1.05), smoking (HR = 1.62, 95%CI: 1.06, 2.47), fever (HR = 2.56, 95%CI: 1.66, 3.95), elevated C-reactive protein (CRP) (HR = 1.02, 95%CI: 1.01, 1.02), rapidly progressive ILD (RP-ILD) (HR = 4.02, 95%CI: 1.89, 8.55), high white blood cell count (WBC) (HR = 1.11, 95%CI: 1.02, 1.21), Krebs von den Lungen-6 (KL-6) (HR = 1.11, 95%CI: 1.06, 1.16), ferritin (≥800 ng/mL) (HR = 6.17, 95%CI: 2.51, 15.20), and lymphocyte count (<1.1×10_9_/L) (HR = 4.88, 95%CI: 1.80, 13.20). Higher PaO_2_ reduced mortality risk (HR = 0.91, 95%CI: 0.86, 0.98). Male, creatine kinase (CK), percent predicted diffusing capacity of the lung carbon monoxide (DLCO%), percent predicted forced vital capacity (FVC%), and erythrocyte sedimentation rate (ESR) showed no significant associations.

**Conclusion:**

Age, smoking, fever, inflammatory markers, and RP-ILD are critical mortality risk factors in MDA5^+^ DM-ILD. Early identification and management of these factors may improve prognosis.

**Systematic review registration:**

http://INPLASY.com, identifier INPLASY202540058.

## Introduction

Dermatomyositis (DM) is an idiopathic inflammatory myopathy (IIM) with inflammatory, immune-mediated organ damage that can involve muscle, skin, joint, heart, and lung. Interstitial lung disease (ILD), as a pulmonary complication, is often associated with DM (1). The anti-melanoma differentiation-associated gene 5 (MDA5) antibody is a specific antibody found in 20%–35% of patients with DM or clinically amyopathic dermatomyositis (CADM). The detection of this antibody has been proved to correlate with a high prevalence of ILD of approximately 60%–80% (2, 3). Approximately half of patients with anti-MDA5 antibody-positive DM with ILD (MDA5^+^ DM-ILD) develop into rapidly progressive ILD (RP-ILD), which usually has a poor prognosis. The majority of deaths occurred in the first 6 months after disease onset, and even after receiving intensive immunosuppressive therapy, the 6-month severe mortality rate in patients with MDA5^+^ DM-ILD is still approximately 50% (2, 4, 5). Although Xie et al. previously conducted a comprehensive analysis of risk factors in the broader MDA5^+^ DM cohort (6), specific data focusing exclusively on the MDA5^+^ DM-ILD subgroup remains scarce. Given that risk factors may vary across clinical phenotypes, existing prognostic models may lack the precision necessary for optimal risk stratification, particularly in the MDA5^+^ DM-ILD subgroup with a higher mortality rate. Therefore, targeted research focusing on this high-risk population is essential. A systematic understanding of the risk factors for mortality in patients with MDA5^+^ DM-ILD can provide assistance for early intervention and management of the disease.

The mortality of MDA5^+^ DM-ILD patients is related to a variety of factors, commonly including malignancies, acute infections, and disease progression, but there is no more relevant research evidence at present. The results of some retrospective studies have found that the mortality of MDA5^+^ DM-ILD patients is associated with age, smoking, Krebs von den Lungen-6 (KL-6), percent predicted forced vital capacity (FVC%), and fever (7–9). However, the findings reported in different studies are not entirely consistent, and some of the results remain controversial due to the lack of pooled evidence.

Therefore, we conducted this systematic review and meta-analysis, aiming to integrate existing evidence and evaluate the association between mortality and potential risk factors in the subgroup of patients with MDA5^+^ DM-ILD. By identifying key prognostic indicators, this study is expected to provide evidence-based basis for the risk stratification of high-risk populations and the optimization of early personalized management strategies.

## Materials and methods

This study was conducted in accordance with the Preferred Reporting Items for Systematic Review and Meta-Analysis (PRISMA) guidelines (10) and was registered at INPLASY (http://INPLASY.com), (registration no.: INPLASY202540058).

### Search strategy

A comprehensive search of English language literature published in PubMed, Embase, Web of Science, and Scopus databases prior to November 18, 2024, was performed. The search terms were as follows: “Dermatomyositis”, “melanoma differentiation-associated gene-5”, “Lung Diseases, Interstitial”, “Lung Diseases, Interstitial”, “MDA5”, “ILD”, etc. (**Supplementary Table 1**).

### Eligibility criteria

The criteria for inclusion were as follows: (1) prospective or retrospective studies; (2) diagnosis of DM was based on the IIM classification criteria of the European League Against Rheumatism/American College of Rheumatology (EULAR/ACR) (11) or Bohan and Peter’s diagnostic criteria (12); (3) diagnosis of ILD was based on established clinical guidelines (13, 14), incorporating respiratory symptoms, physical examination findings, abnormalities on high-resolution computed tomography (HRCT), and pulmonary function test results through multidisciplinary evaluation; (4) RP-ILD was defined as a progressive deterioration of interstitial changes on radiological assessment of HRCT examination within 3 months, with or without concomitant clinical symptom deterioration (15–17) (**Supplementary Table 2**); (5) hazard ratios (HR) and 95% confidence intervals of mortality risk factors in MDA5^+^ DM-ILD were obtained by the Cox proportional hazards regression model; (6) English literature.

The criteria for exclusion were as follows: (1) duplicate literature; (2) case report, conference abstract, review or meta-analysis, animal or cell study, comment or letter, etc.; (3) studies not related to MDA5^+^ DM-ILD; (4) mortality of MDA5^+^ DM-ILD is not the outcome event; (5) inability to extract data; (6) literature not in English.

### Variable selection and subgroup analysis

YL and WY searched the databases according to the process of PRISMA guidelines, read the abstracts and full text of the literature related to MDA5^+^ DM-ILD, and selected the risk factors obtained from univariate Cox proportional hazards regression analysis. Risk factors to be included in the pooled analysis were required to satisfy that at least two of the included literatures were able to extract relevant data. Subgroup analyses were performed for different risk factors. Each risk factor was regarded as an independent meta-analysis rather than using the traditional subgroup analysis method. Due to the significant heterogeneity among the subgroups, we did not perform pooled analyses for the risk factors among the subgroups.

### Quality assessment (risk of bias) and data extraction

YL and WY independently screened all studies, with conflicts resolved by an arbitrator (JJ). The quality of the included studies was assessed using the Newcastle–Ottawa Scale (NOS), a semiquantitative scoring system (18). The NOS is a widely utilized tool for assessing the quality of case–control and cohort studies. It evaluates study quality through three major modules comprising a total of eight items. These items specifically address the study population selection, comparability, and assessment of exposure/outcome. The total score of NOS ranges from 0 to 9 “stars,” with higher scores indicating higher quality of included studies: 7–9 “stars” signifying high quality, 4–6 “stars” indicating moderate quality, and 0–3 “stars” reflecting low quality.

The literature quality assessment was independently completed by SZ and XH. Relevant data information about the included literature was independently extracted by YL and WY, including authors’ surnames, publication year, country, type of study, age, male, proportion of smokers, follow-up time, FVC%, DLCO%, study sample size and number of deaths, risk factors reported in the study, hazard ratios (HR), 95% confidence intervals (CI), and study outcome.

### Study outcome and data synthesis

Given that disease-specific mortality is conceptually a subset of all-cause mortality, and to ensure a uniform endpoint definition for pooled analysis in this meta-analysis, we regarded all reported mortality events (including disease-specific mortality) as contributing to all-cause mortality events in our analysis. Therefore, the composite effect estimate from this meta-analysis reflects the risk of all-cause mortality. HR and 95% CI of risk factors were collected as statistical effect sizes, and Cochran’s Q statistic and inconsistency value (I^2^) was used to test for heterogeneity of the included studies. If *P* < 0.05 and I^2^ ≥ 50%, heterogeneity was significant, and pooled analyses were performed using the random effects model and DerSimonian–Laird (DL) method. Otherwise, the fixed effects model and inverse variance (IV) method were used. Subgroup analyses were executed for different risk factors. Excluding one category of study at a time method was utilized for sensitivity analysis. If there was no significant effect on the results after excluding a study, it means that our results were stable and reliable. Egger’s test was used to assess publication bias, and trim-and-fill method funnel plots were used to jointly determine the reliability of the articles. Statistical analyses were computed using the meta package in Stata 16.0 software, with statistical significance at *P* < 0.05.

## Result

**Figure 1:**
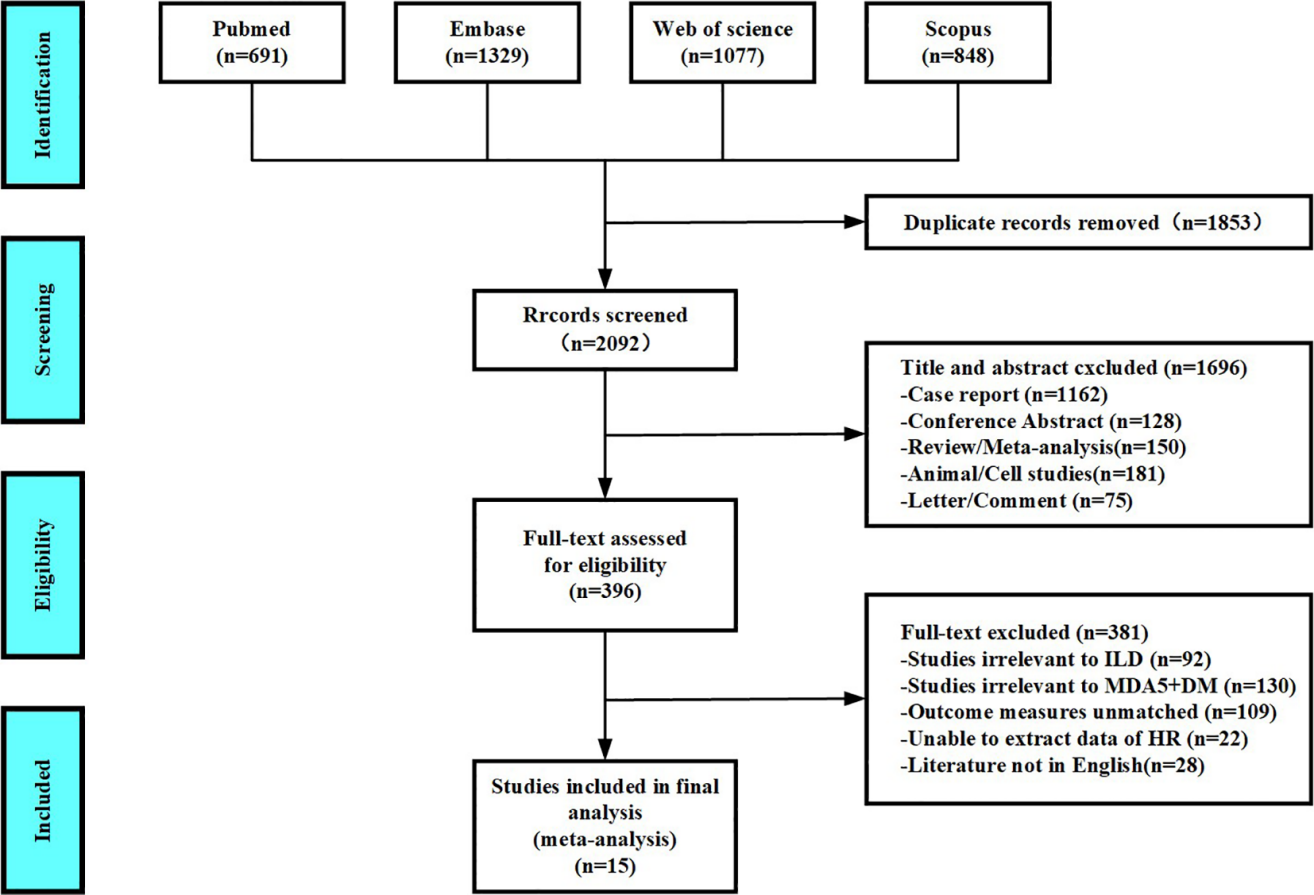
Diagram of the preferred reporting items for systematic review and meta-analysis (PRISMA).

According to the process of PRISMA guidelines, abstracts and full texts of 3,945 MDA5^+^ DM-ILD-related studies were critically evaluated. Through stratified exclusion criteria, we removed 1,853 duplicate studies, 1,162 case reports, 128 conference abstracts, 150 reviews or meta- analysis, 181 animal or cell studies, 75 letters or comments, 92 not related to ILD, 130 not related to MDA5^+^ DM, 109 mismatched outcome events, 22 unable to extract data, and 28 non-English literatures. A total of 15 studies were finally included (**Figure 1**). We performed a pooled analysis of risk factors for mortality in 1,153 patients from the 15 final included studies. Of all the included studies, 14 were retrospective study designs and 1 was a prospective study. The countries involved in the study were China (n=10), Japan (n=4), and France (n=1). The risk factors of MDA5^+^ DM-ILD mortality were pooled and analyzed, including age (1, 3, 4, 9, 19–23), male (1, 7, 9, 22, 24), smoking (2, 19, 22), fever (7, 19, 24, 25), C-reactive protein(CRP) (1, 8, 9, 19, 21, 22), creatine kinase (CK) (7, 19, 22), percent predicted diffusing capacity of the lung carbon monoxide (DLCO%) (1, 8, 19), PaO_2_ (3, 8, 19), FVC% (1, 8, 19), RP-ILD (2, 22, 23), erythrocyte sedimentation rate (ESR) (1, 19, 22), white blood cell count (WBC) (19, 22), KL-6 (7, 9), ferritin (≥800 ng/mL) (3, 24), and lymphocyte count (< 1.1×10^9^/L) (24, 26). The content characteristics of all studies are shown in **Table 1**. After NOS literature quality evaluation, all 15 studies were recognized as high quality studies (score ≥7 stars), among which two studies received full marks (9 stars), and the remaining studies had a score range of 7–8 stars (**Supplementary Table 3**). High-quality research significantly improved the robustness of the results.

**Table 1:** Clinical characteristics and univariate Cox regression results for mortality risk factors in each included study.

No	Author, year	Country	Study type	Age (year)	Male N (%)	Smoking N (%)	Follow-up time	FVC%	DLCO%	nTotal/nDeath	Risk factor (survival)	HR	Lower limit	Higher limit	Outcome
1	Mao M, et al., 2020 (20)	China	Retrospective	NA	17 (42.5)	NA	3m	NA	NA	40/22	Age	1.10	1.02	1.17	All-cause mortality
2	Li Y, et al., 2023 (19)	China	Retrospective	53.0* [32.0-77.0]	17 (41.5)	13 (31.7)	NA	66.09 [36.1-86.2]	58.45 [8.2-81.7]	41/26	Age	1.050	1.013	1.088	All-cause mortality
											Smoking	1.611	0.714	3.633	
											Fever	2.848	1.251	6.485	
											CRP	1.020	1.001	1.039	
											CK	1.001	0.997	1.005	
											DLCO%	1.026	0.969	1.086	
											PaO_2_	0.957	0.925	0.989	
											FVC%	1.064	0.992	1.141	
											ESR	0.990	0.971	1.009	
											WBC	1.078	0.940	1.235	
3	Xu W, et al., 2021 (4)	China	Retrospective	50 [42-58]	54 (35.5)	NA	6m	60.8 [45.9-72.5]	NA	152/62	Age	1.04	1.02	1.07	All-cause mortality
4	Yamaguchi K, et al., 2022 (21)	Japan	Retrospective	55 ± 12^#^	11 (32)	NA	1024d [20-1095]	NA	NA	34/8	Age	1.09	1.02	1.16	Disease-specific mortality
											CRP	1.98	1.31	2.99	
5	Fukada A, et al., 2024 (7)	Japan	Retrospective	53 [46-65]	21 (26.6)	31 (39.2)	27.3m [3.5-64.4]	78.0 [64.4-93.3]	67.2 [55.3-85.3]	79/20	Sex	1.631	0.650	4.092	All-cause mortality
											Fever	3.390	1.230	9.337	
											CK	1.088	0.954	1.240	
											KL-6	1.108	1.049	1.171	
6	Zhou W, et al., 2024 (22)	China	Retrospective	58.17 ± 9.07	12 (29.3)	9 (22.0)	442.19 ± 623.49d	NA	NA	41/35	Age	1.021	0.981	1.063	All-cause mortality
											Sex	1.173	0.571	2.411	
											Smoking	0.921	0.401	2.111	
											CRP	1.011	1.001	1.022	
											CK	1.000	0.996	1.005	
											RP-ILD	1.829	0.928	3.604	
											ESR	1.002	0.988	1.016	
											WBC	1.132	1.016	1.261	
7	Li Y, et al., 2020 (26)	China	Retrospective	56.0 ± 9.1	6 (21.4)	NA	NA	79.5 ± 17.1	60 ± 15.6	28/9	Lymphocyte count (< 1.1×10^9^/L)	4.80	1.22	18.92	Disease-specific mortality
8	Lian X, et al., 2023 (23)	China	Prospective	52 [16-73]	75 (34.7)	NA	NA	63.6 ± 11.2	41.2 ± 9.2	216/85	Age	1.027	1.002	1.053	All-cause mortality
											RP-ILD	5.072	2.653	9.695	
9	Waseda Y, et al., 2022 (8)	Japan	Retrospective	53.6 ± 13.5	7 (35)	19 (95)	1242.9 ± 1324.8 d	81.9 ± 19.6	NA	20/7	CRP	1.594	0.943	2.694	All-cause mortality
											DLCO%	0.988	0.942	1.037	
											PaO_2_	0.886	0.805	0.975	
											FVC%	0.937	0.886	0.991	
10	Liu H, et al., 2024 (2)	China	Retrospective	50.0 [44.0-56.5]	61 (33.3)	32 (17.5)	23.4m [1-57]	NA	NA	183/53	Smoking	2.21	1.20	4.08	All-cause mortality
											RP-ILD	6.66	3.76	11.80	
11	Gui X, et al., 2021 (1)	China	Retrospective	54.0 [47.0-62.5]	30 (41.1)	NA	12m	63.7 [47.2-78.8]	55.7 [45.3-67.9]	73/38	Age	1.044	0.987	1.105	All-cause mortality
											Sex	1.304	0.674	2.521	
											CRP	1.021	1.007	1.036	
											DLCO%	1.004	0.945	1.066	
											FVC%	1.014	0.966	1.065	
											ESR	1.009	0.995	1.023	
12	Bay P, et al., 2022 (25)	France	Retrospective	51 ± 12	17 (33.3)	NA	77d [38-264]	62 [46-76]	40 [32-47]	51/31	Fever	1.9	0.9	4.2	All-cause mortality
13	Ye Y, et al., 2019 (9)	China	Retrospective	50.02 ± 9.34	17 (25)	NA	23.6m	NA	NA	68/18	Age	1.022	0.983	1.052	Disease-specific mortality
											Sex	0.602	0.293	1.728	
											CRP	1.020	0.853	1.232	
											KL-6	1.122	1.031	1.227	
14	Fujisawa T, et al., 2019 (3)	Japan	Retrospective	54.0 [31-80]	7 (23)	12 (40)	1.01yrs [0.02-18.8]	72.3 [36.6-125.5]	NA	30/10	Age	1.04	0.979	1.11	Disease-specific Mortality
											PaO_2_	0.87	0.783	0.946	
											Ferritin (≥ 800 ng/mL)	5.42	1.35	35.9	
15	Zhao S, et al., 2022 (24)	China	Retrospective	NA	27 (27.8)	NA	NA	NA	NA	97/36	Sex	2.10	0.94	4.68	All-cause mortality
											Fever	2.71	1.08	6.78	
											Ferritin (≥ 800 ng/mL)	6.53	2.22	19.18	
											Lymphocyte count (< 1.1×10^9^/L)	4.97	1.17	21.10	

*Median [interquartile range]; #, mean ± standard deviation; %, percentage of predicted value; NA, not applicable.

MDA5, melanoma differentiation-associated gene 5; DM, dermatomyositis; ILD, interstitial lung disease; DLCO%, percent predicted diffusing capacity of the lung carbon monoxide; FVC%, percent predicted forced vital capacity; HR, hazard ratio; CI, confidence interval; CRP, C-reactive protein; CK, creatine kinase; ESR, erythrocyte sedimentation rate; WBC, white blood cell count; KL-6, Krebs von den Lungen-6; RP-ILD, rapidly progressive interstitial lung disease.

### Pooled analysis of the effect of age on mortality in MDA5^+^ DM-ILD

**Figure 2:**
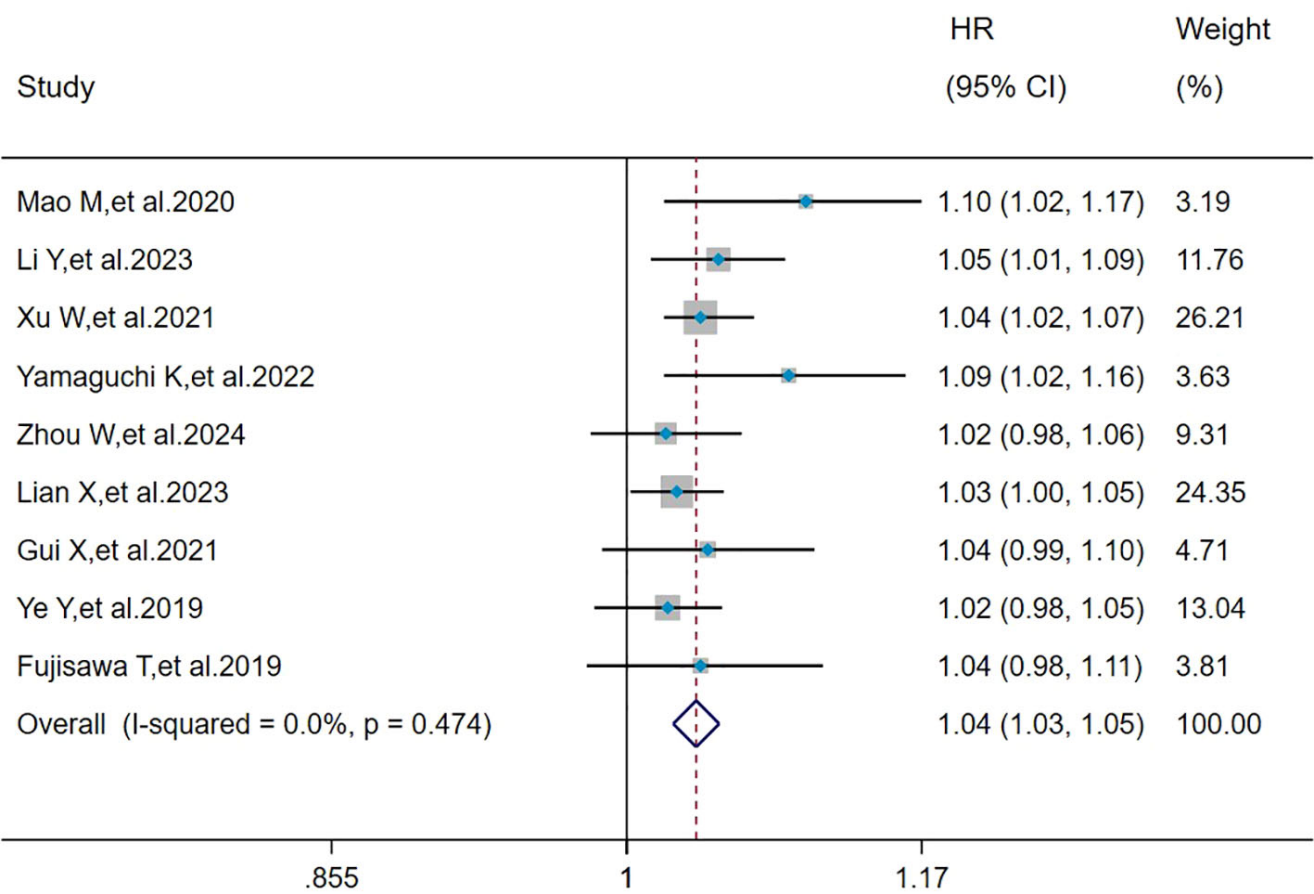
Forest plot: hazard ratio of age for mortality in MDA5^+^ DM-ILD. HR, hazard ratio; CI, confidence interval.

The heterogeneity results indicated that among the risk factors for mortality, age (I^2^ = 0%, *P =* 0.474) showed no significant heterogeneity. Using the fixed-effects model and IV method for analysis, the pooled results revealed that age (HR = 1.04, 95%CI: 1.03, 1.05, *P* < 0.001) was a risk factor for mortality in patients with MDA5^+^ DM-ILD (**Figure 2**).

### Pooled analysis of the effects of male, smoking, and fever on mortality in MDA5^+^ DM-ILD

**Figure 3:**
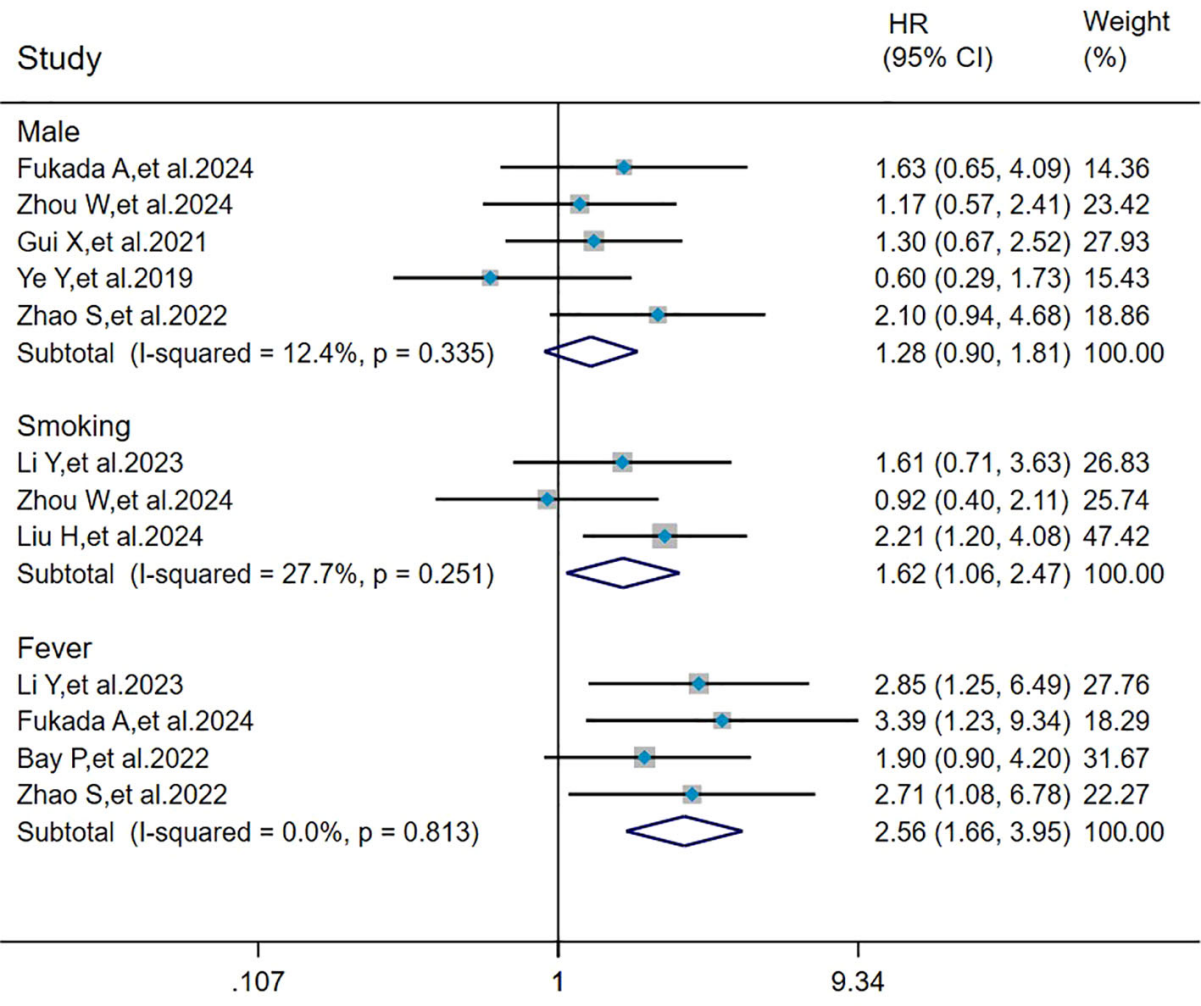
Forest plot: pooled hazard ratio of male, smoking, and fever for mortality in MDA5^+^ DM-ILD. HR, hazard ratio; CI, confidence interval.

The heterogeneity results indicated that among the risk factors for mortality, male (I^2^ = 12.4%, *P* = 0.335), smoking (I^2^ = 27.7%, *P* = 0.251), and fever (I^2^ = 0%, *P* = 0.813) showed no significant heterogeneity. Using the fixed-effects model and IV method for analysis, the pooled results showed that smoking (HR = 1.62, 95%CI: 1.06, 2.47, *P =* 0.025) and fever (HR = 2.56, 95%CI: 1.66, 3.95, *P* < 0.001) were the risk factors for mortality in patients with MDA5^+^ DM-ILD, whereas male (HR = 1.28, 95%CI: 0.90, 1.81, *P =* 0.171) had no effect on mortality (**Figure 3**).

### Pooled analysis of the effects of CRP, CK, and DLCO% on mortality in MDA5^+^ DM-ILD

**Figure 4:**
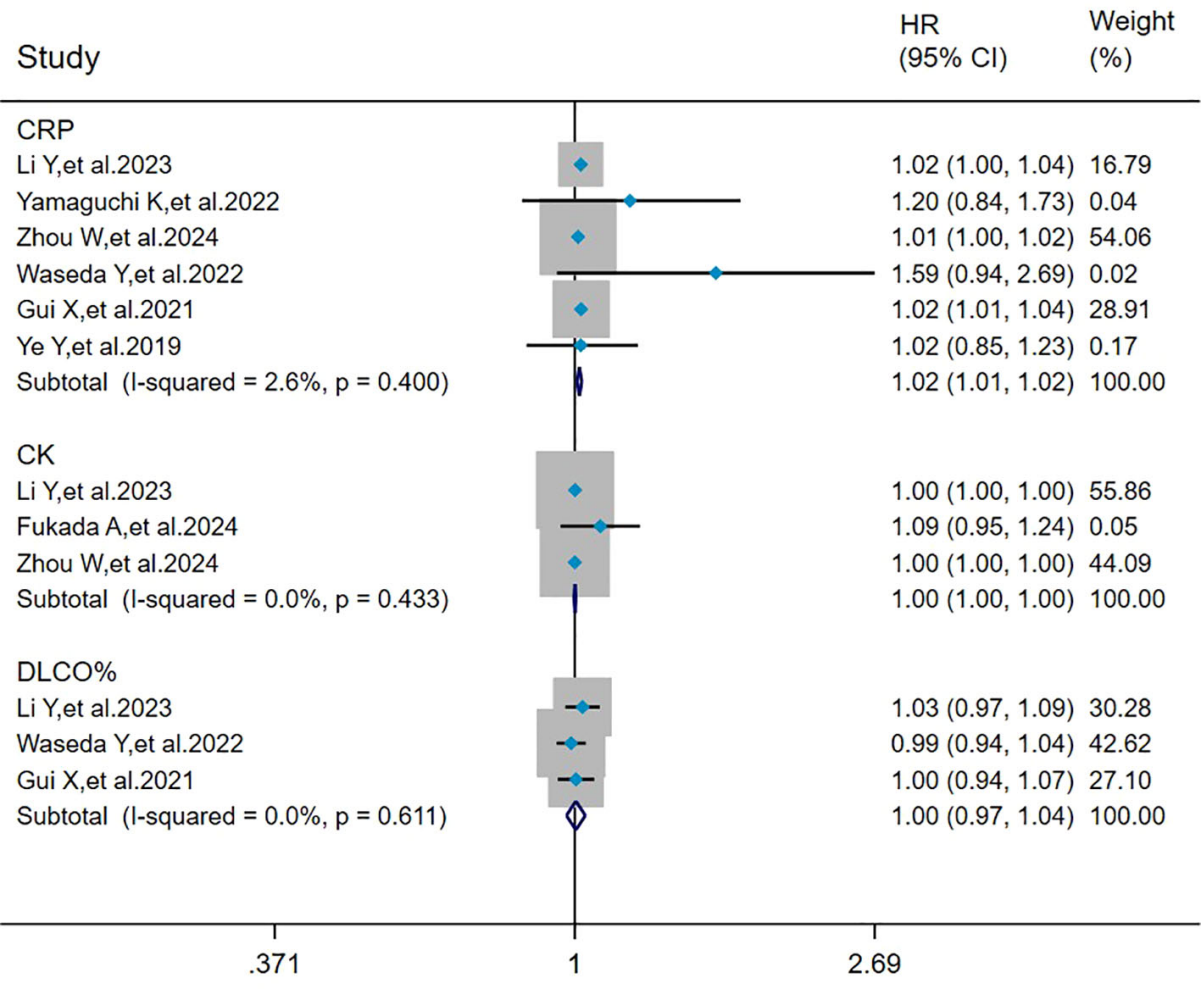
Forest plot: pooled hazard ratio of CRP, CK, and DLCO% for mortality in MDA5^+^ DM-ILD. CRP, C-reactive protein; CK, creatine kinase; DLCO%, percent predicted diffusing capacity of the lung carbon monoxide; HR, hazard ratio; CI, confidence interval.

The heterogeneity results indicated that among the risk factors for mortality, CRP (I^2^ = 2.6%, *P* = 0.400), CK (I^2^ = 0%, *P* = 0.433), and DLCO% (I^2^ = 0%, *P* = 0.611) showed no significant heterogeneity. Using the fixed-effects model and IV method for analysis, the pooled results showed that CRP (HR = 1.02, 95%CI: 1.01, 1.02, *P* < 0.001) was a risk factor for mortality in patients with MDA5^+^ DM-ILD, whereas CK (HR = 1.00, 95%CI: 1.00, 1.00, *P* = 0.693) and DLCO% (HR = 1.00, 95%CI: 0.97, 1.04, *P* = 0.817) had no effect on mortality in MDA5^+^DM-ILD (**Figure 4**).

### Pooled analysis of the effect of PaO_2_, FVC%, and RP-ILD on mortality in MDA5^+^ DM-ILD

**Figure 5:**
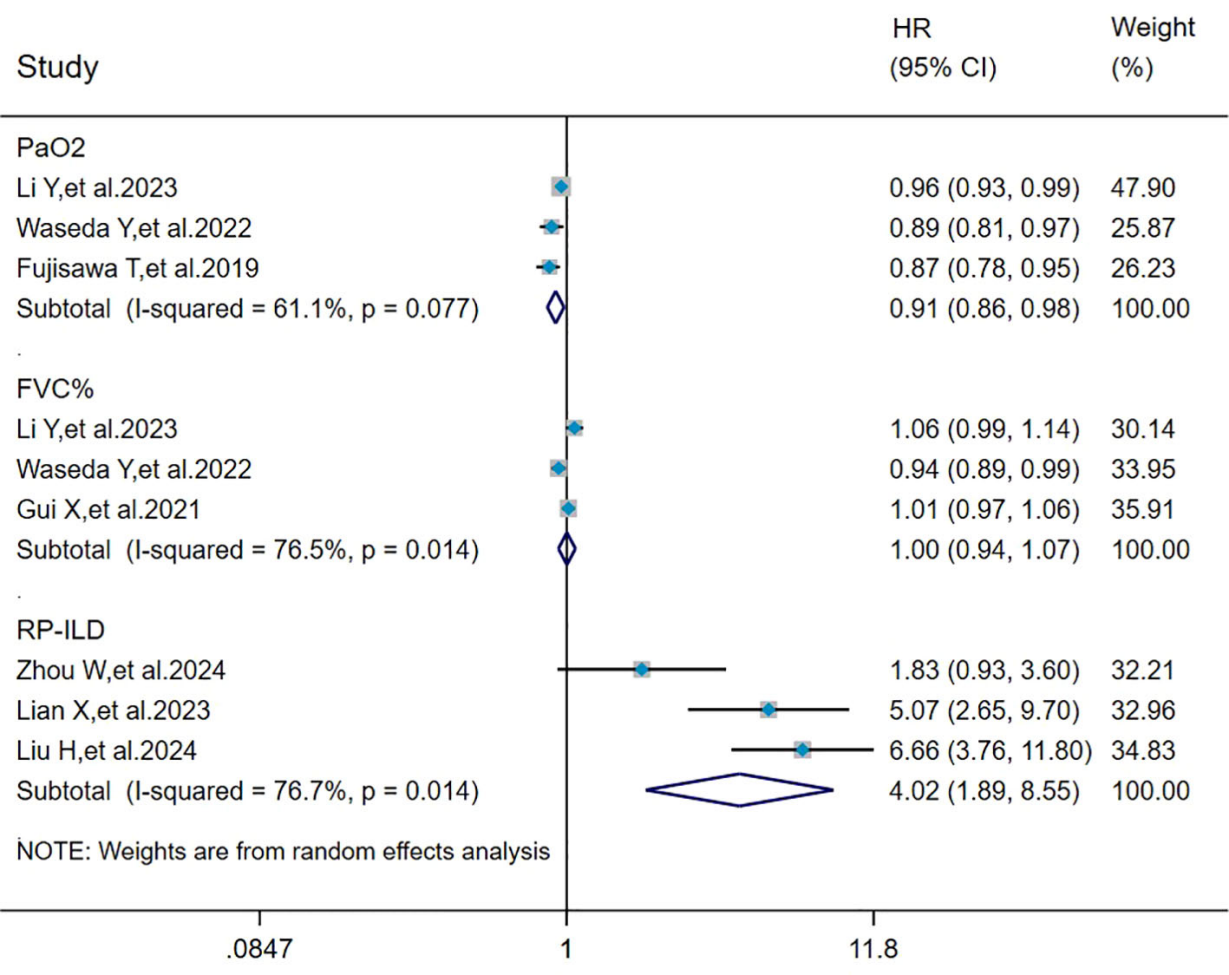
Forest plot: pooled hazard ratio of PaO_2_, FVC%, and RP-ILD for mortality in MDA5^+^ DM-ILD. FVC%, percent predicted forced vital capacity; RP-ILD, rapidly progressive interstitial lung disease; HR, hazard ratio; CI, confidence interval.

The heterogeneity results indicated that among the risk factors for mortality, PaO_2_ (I^2^ = 61.1%, *P* = 0.077), FVC% (I^2^ = 76.5%, *P* = 0.014), and RP-ILD (I^2^ = 76.7%, *P* = 0.014) showed significant heterogeneity. Analyzed by the random effects model and DL method, the pooled results showed that RP-ILD (HR = 4.02, 95%CI: 1.89, 8.55, *P* < 0.001) was a risk factor for mortality in patients with MDA5^+^ DM-ILD, and PaO_2_ (HR = 0.91, 95%CI: 0.86, 0.98, *P* = 0.010) was a protective factor against MDA5^+^ DM-ILD mortality, whereas FVC% (HR = 1.00, 95%CI: 0.94, 1.07, *P =* 0.964) had no effect on mortality in MDA5^+^ DM-ILD (**Figure 5**).

### Pooled analysis of the effects of ESR, WBC, and KL-6 on mortality in MDA5^+^ DM-ILD

**Figure 6:**
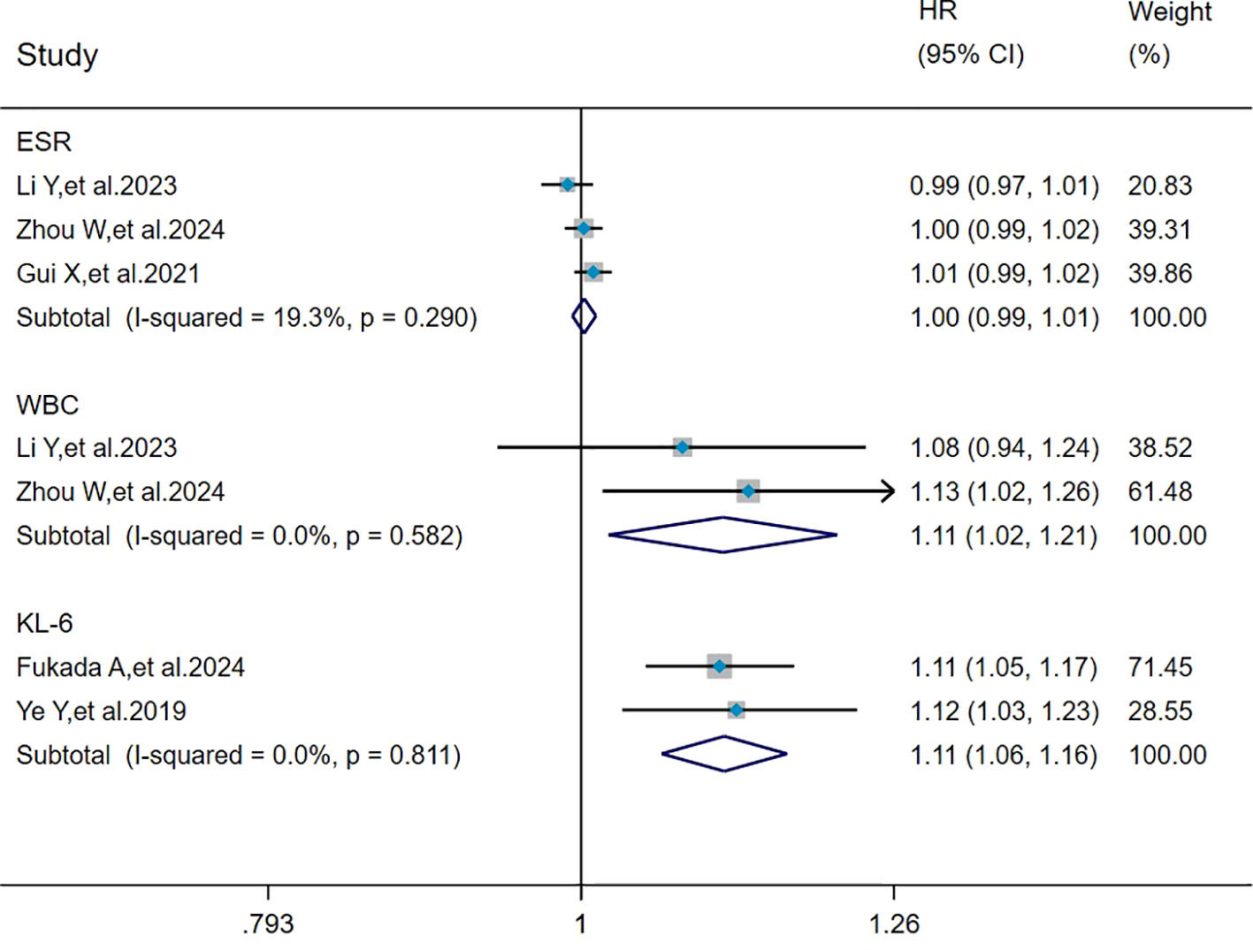
Forest plot: pooled hazard ratio of ESR, WBC, and KL-6 for mortality in MDA5^+^ DM-ILD. ESR, erythrocyte sedimentation rate; WBC, white blood cell count; KL-6, Krebs von den Lungen-6; HR, hazard ratio; CI, confidence interval.

The heterogeneity results indicated that among the risk factors for mortality, ESR (I^2^ = 19.3%, *P* = 0.290), WBC (I^2^ = 0%, *P* = 0.582), and KL-6 (I^2^ = 0%, *P* = 0.811) showed no significant heterogeneity. Using the fixed-effects model and IV method for analysis, the pooled results showed that WBC (HR = 1.11, 95%CI: 1.02, 1.21, *P* = 0.015) and KL-6 (HR = 1.11, 95%CI: 1.06, 1.16, *P* < 0.001) were the risk factors for mortality in patients with MDA5^+^ DM-ILD whereas ESR (HR = 1.00, 95%CI: 0.99, 1.01, *P* = 0.613) had no effect on mortality (**Figure 6**).

### Pooled analysis of the effect of ferritin (≥ 800 ng/mL) and lymphocyte count (<1.1×10^9^/L) on mortality in MDA5^+^ DM-ILD

**Figure 7:**
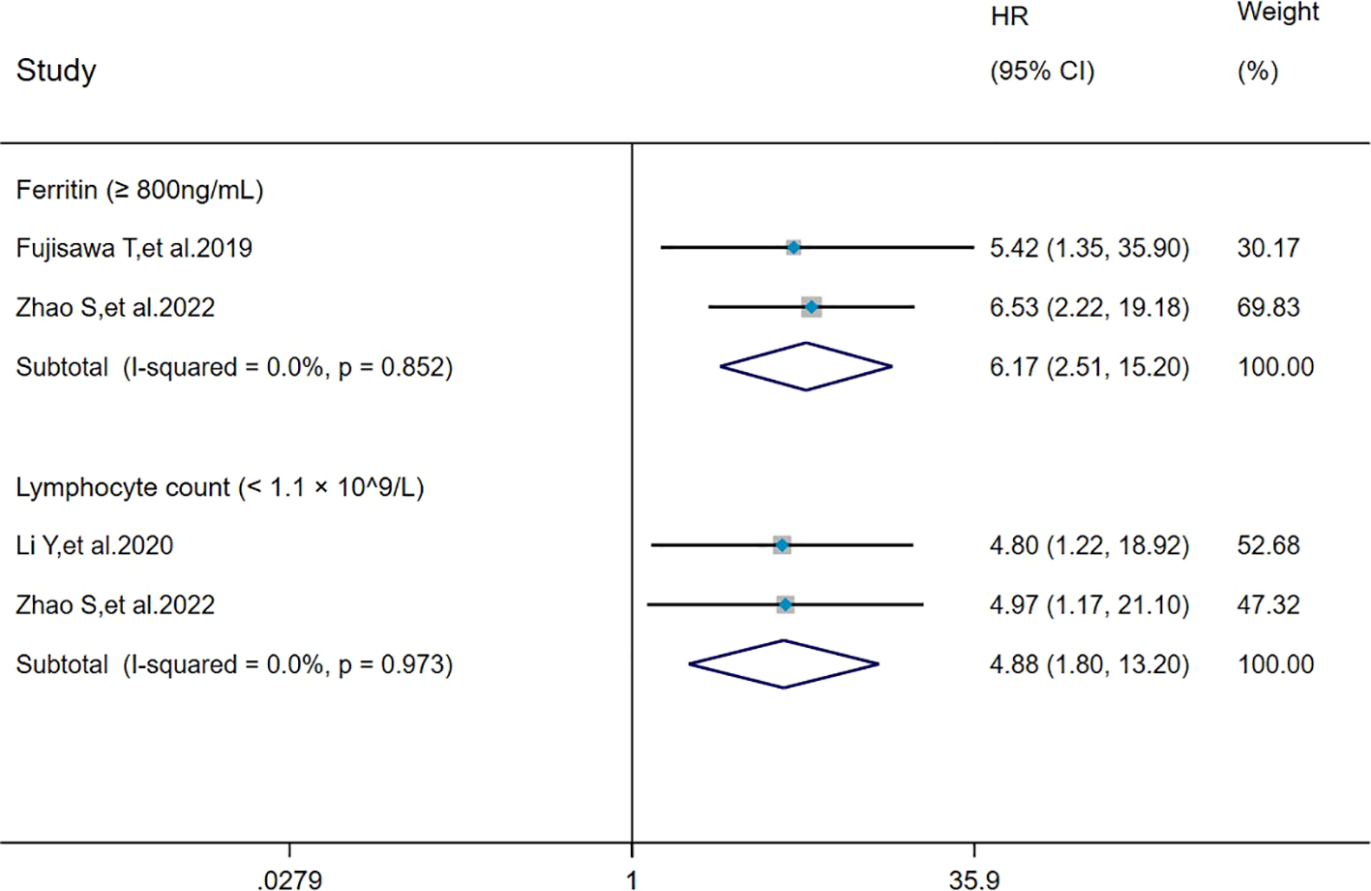
Forest plot: pooled hazard ratio of ferritin (≥800 ng/mL) and lymphocyte count (< 1.1×10^9^/L) for mortality in MDA5^+^ DM-ILD. HR, hazard ratio; CI, confidence interval.

The heterogeneity results indicated that among the risk factors for mortality, ferritin (≥800 ng/mL) (I^2^ = 0%, *P* = 0.852) and lymphocyte count (< 1.1 × 10^9^/L) (I^2^ = 0%, *P* = 0.973) showed no significant heterogeneity. The analysis was performed using the fixed-effects model and IV method, and the pooled results showed that ferritin (≥800 ng/mL) (HR = 6.17, 95%CI: 2.51, 15.20, *P* < 0.001) and lymphocyte count (<1.1×10^9^/L) (HR = 4.88, 95%CI: 1.80, 13.20, *P =* 0.002) were risk factors for mortality in patients with MDA5^+^ DM-ILD (**Figure 7**).

### Sensitivity analysis and publication bias

**Figure 8:**
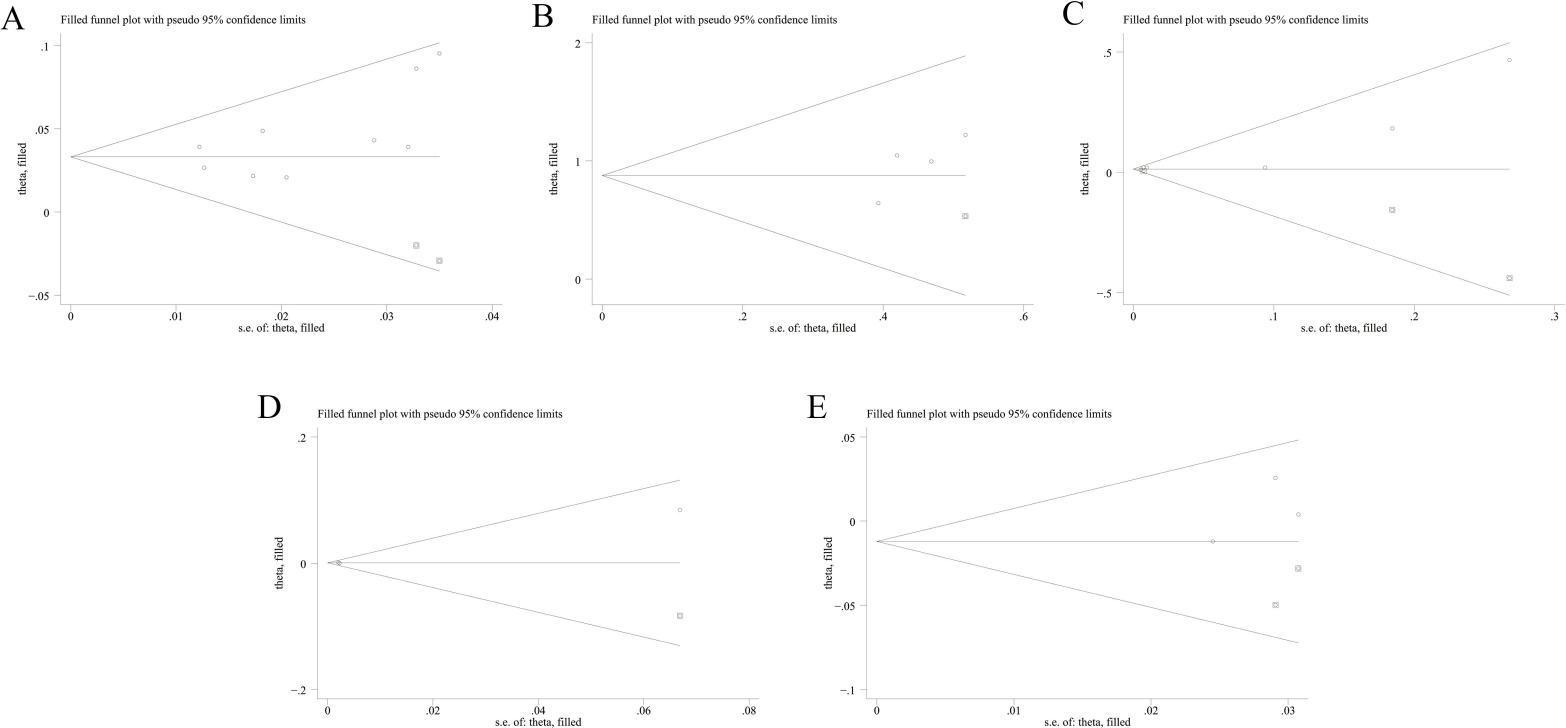
Funnel plots of trim-and-fill analysis for hazard ratio predicting mortality of MDA5^+^ DM-ILD (**(A)** age, **(B)** fever, **(C)** CRP, **(D)** CK, **(E)** DLCO%). MDA5, melanoma differentiation-associated gene 5; DM, dermatomyositis; ILD, interstitial lung disease; CRP, C-reactive protein; CK, creatine kinase; DLCO%, percent predicted diffusing capacity of the lung carbon monoxide.

The sensitivity analysis indicated that our findings were stable (**Supplementary Figures 1**-**15**). The results of heterogeneity analysis showed that there was no significant heterogeneity in the included literature for all factors, except for PaO_2_, FVC%, and RP-ILD for which there was significant heterogeneity in the included literature. Egger’s test indicated no significant publication bias (P > 0.05) for factors with ≥3 studies (**Table 2**). Subsequently, all pooled studies on risk factors associated with MDA5^+^ DM-ILD mortality were adjusted by the trim-and-fill method. The analyses for age (n = 2), fever (n = 1), CRP (n = 3), CK (n = 1), and DLCO% (n = 2) showed that after supplementing corresponding literatures, the funnel plots were symmetrical and the statistical results remained unchanged (**Figures 8A–E**). Male, smoking, PaO_2_, FVC%, RP-ILD, and ESR had symmetrical funnel plots (**Supplementary Figures 16A-F**). For risk factors with only two studies (WBC, KL-6, ferritin (≥800 ng/mL), lymphocyte count (<1.1×10_9_/L)), Egger’s test was not applicable. However, although the number of included studies was small, the combination of sensitivity analysis and the results of the trim-and-fill method funnel plot indicated that publication bias was not considered in the pooled results (**Supplementary Figures 12**-**15**, **16G-J**). This indicates that all the studies we included did not consider publication bias.

**Table 2:** Sensitivity analysis, Egger’s test, MetaTrim-filled study, funnel plot, and publication bias of included studies with different groups.

Risk factors	Sensitivity analysis (excluded study)	Included items	Egger’s test		MetaTrim-filled study	Funnel plot	Publication bias
			t	*P*			
Age	0	9	1.99	0.087	2	Symmetry	No
Male	0	5	-0.23	0.831	0	Symmetry	No
Smoking	0	3	-1.65	0.346	0	Symmetry	No
Fever	0	4	2.10	0.170	1	Symmetry	No
CRP	0	6	2.41	0.073	3	Symmetry	No
CK	0	3	2.82	0.217	1	Symmetry	No
DLCO%	0	3	1.02	0.493	2	Symmetry	No
PaO_2_	0	3	-7.73	0.082	0	Symmetry	No
FVC%	0	3	0.39	0.763	0	Symmetry	No
RP-ILD	0	3	-1.61	0.354	0	Symmetry	No
ESR	0	3	-2.15	0.277	0	Symmetry	No
WBC	0	2	NA	NA	0	Symmetry	No
KL-6	0	2	NA	NA	0	Symmetry	No
Ferritin (≥800 ng/mL)	0	2	NA	NA	0	Symmetry	No
Lymphocyte count (<1.1×10^9^/L)	0	2	NA	NA	0	Symmetry	No

NA, not applicable; CRP, C-reactive protein; CK, creatine kinase, DLCO%, percent predicted diffusing capacity of the lung carbon monoxide; FVC%, percent predicted forced vital capacity; RP-ILD, rapidly-progressive interstitial lung disease; ESR, erythrocyte sedimentation rate; WBC, white blood cell count; KL-6, Krebs von den Lungen-6.

## Discussion

In recent years, the exploration of risk factors for mortality in MDA5^+^ DM-ILD has become a hot research topic in the medical field. Given that patients with MDA5^+^ DM-ILD still exhibit an alarmingly high short-term mortality rate of 50% despite early detection and aggressive treatment (4), and considering that prognostic factors may differ across clinical phenotypes, prognostic models derived from pooled analyses of general MDA5^+^ DM cohorts (with or without ILD) may lack the precision required for optimal risk stratification of this high-mortality subgroup. Therefore, although Xie et al. (6) have conducted a comprehensive meta-analysis on prognostic factors in MDA5^+^ DM (regardless of ILD status), it is imperative to perform targeted research specifically focused on the high-mortality MDA5^+^ DM-ILD subpopulation. Moreover, some controversial results were observed across different studies. Identifying the risk factors for mortality in MDA5^+^ DM-ILD not only contributes to a more systematic and comprehensive understanding of the disease but also serves as a foundation for developing clinical early intervention strategies and disease management, which is both scientifically valuable and clinically significant. Therefore, this study focused on patients with MDA5^+^ DM particularly combined with ILD, aiming to explore novel biomarkers and potential cutoff values and conduct a pooled analysis of these potential risk factors. It was found that age, smoking, fever, CRP, RP-ILD, WBC, KL-6, ferritin (≥800 ng/mL), and lymphocyte count (<1.1×10^9^/L) were associated with mortality in MDA5^+^ DM-ILD.

Pooled analyses showed that age was a risk factor for mortality in MDA5^+^ DM-ILD patients. For every decade increase in age, the mortality rate of patients with MDA5^+^ DM rises 1.5-fold (6). Yamaguchi et al. reported that older patients with more insidious clinical symptoms than younger patients had a higher mortality rate and that combined immunosuppressive therapy was usually ineffective for RP-ILD in older patients (27). This phenomenon may correlate with age-related declines in organ function and drug metabolism capacity. In addition, current or former smoking status was associated with high mortality risk. It has long been known that smoking causes lung damage. One study found that the development of ILD was significantly associated with a significantly higher likelihood of exposure to tobacco smoke in smokers (28). The underlying mechanism involves an inflammatory cascade activation across multiple immune cell types, perpetuating a vicious cycle of inflammatory cell recruitment (29). This suggests that smoking not only contributes to ILD pathogenesis but also exacerbates mortality risk. Fever and RP-ILD were also risk factors for mortality in MDA5^+^ DM-ILD. Fever has been identified as an independent predictor of RP-ILD in MDA5^+^ DM patients (30). Niu et al. incorporated fever into a clinical prediction model demonstrating high accuracy for mortality risk stratification (31), aligning with our findings that febrile states may accelerate disease progression. Wang et al. highlighted temporal patterns in RP-ILD outcomes, with more than 90% of RP-ILD and 84% of death occurring within the first 6 months after the onset of the disease (32). This further demonstrates the serious threat of RP-ILD to patients’ lives in early stages of the disease. Therefore, clinical attention should be paid to patients with fever or RP-ILD, and timely therapeutic interventions are essential to improve the survival of patients. Elevated CRP levels inversely correlated with survival. It has been noted that CRP <0.8 mg/dL is positively correlated with survival in patients with MDA5^+^ DM (6), supporting its prognostic utility. Some studies have pointed out that increased WBC is independently correlated with decreased lung function, which may be a simple and easily obtained indicator reflecting changes in lung function (33). A finding extended by our study showed that elevated WBC levels were associated with an increased risk of mortality in MDA5^+^ DM-ILD, suggesting that WBC may be indicative of poor prognostic outcomes. High serum KL-6 level was also a risk factor for poor patient prognosis. Ye et al. analyzed that the optimal critical value of KL-6 for predicting survival was 792 U/mL, with higher values reflecting more severe pulmonary lesions and functional impairment (9). This suggests that KL-6 levels are valuable in assessing the risk of mortality in patients with MDA5^+^ DM-ILD. Compared with previous studies (6), these newly identified risk factors (smoking, fever, and elevated WBC) were not systematically evaluated in prior research. Moreover, we further identified critical thresholds for mortality risk stratification, including ferritin (≥800 ng/mL) and lymphocyte count (<1.1 × 10_9_/L). Ferritin (≥800 ng/mL) was found to be a risk factor for mortality in MDA5^+^ DM-ILD patients in this study. Consistent with the results of this study, Gono et al. found that ferritin level (≥828 ng/mL) was a poor prognostic factor for MDA5^+^ DM combined with RP-ILD (34). Ferritin was an indicator of the RP-ILD related to IIM, which indicated the systemic inflammatory state and the risk of multiple-organ injury, and was an important biomarker to predict the mortality of IIM-ILD (35). This showed the importance of detecting ferritin levels in assessing the condition and prognosis of MDA5^+^ DM-ILD patients. Lymphocyte count (<1.1×10^9^/L) similarly predicted mortality, with multicenter data showing inverse correlations between lymphocyte counts and 1-month survival (36). Subset analyses further implicated that CD3^+^ CD8^+^ T-lymphocyte count (≤31.38/μL) was associated with 180-day mortality in patients with DM-ILD (37). These findings were consistent with the results of our study, providing mechanistic insights for targeted therapies. Notably, the pooled analysis of this study found that PaO_2_ was a protective factor for mortality. Higher PaO_2_ levels conferred protection against mortality, consistent with Xie et al.’s findings (6). This protective effect may stem from enhanced pulmonary gas exchange efficiency and consequent preservation of organ function.

A number of factors that may not be associated with mortality in MDA5^+^ DM-ILD were also identified through the pooled analysis of this study. While some studies suggest that male sex correlates with poorer prognosis in MDA5^+^ DM patients (38), this association was not observed in our study, probably due to the low proportion of male patients in our study and gender distribution bias. Similarly, elevated CK levels did not predict mortality in MDA5^+^ DM-ILD patients. Previous studies have suggested that elevated CK was a risk factor for poor prognosis in MDA5^+^ DM (6). However, considering that death in patients with MDA5^+^ DM-ILD is influenced by a combination of factors and that patients face a very high risk of death if the lung lesions progress rapidly, leading to severe pulmonary dysfunction, respiratory failure, etc., these factors may obscure the relationship between CK and death. FVC% and DLCO%, as key lung function indices, are routinely combined in clinical practice to comprehensively evaluate lung function (39). Pooled data from Xie et al. (6) showed no significant correlation between DLCO% and the risk for mortality in patients with MDA5^+^ DM, which is in line with our results. However, they observed a threefold increased risk in patients with FVC% <50%. Previous studies that included all patients with MDA5^+^ DM (with or without ILD) have certain limitations and may not be sufficient to conduct the optimal risk stratification for the ILD subgroup with a higher risk of death. Moreover, the rapid deterioration of MDA5^+^ DM-ILD may have led to death before a significant decline in lung function. A retrospective study of DM patients by Dong Jin Go et al. showed that the continuous increase of ESR predicted a worse overall mortality (40). In our findings, the increase of ESR had no effect on mortality in MDA5^+^ DM-ILD, possibly attributable to baseline population differences.

Indeed, exploring risk factors for mortality in patients with MDA5^+^ DM-ILD is a long-term endeavor, and there are still some important contents that have not been developed yet. On the one hand, the relationship between biomarkers with potential diagnostic value and mortality in MDA5^+^ DM-ILD remains undefined, such as lactate dehydrogenase (LDH), antinuclear antibody titers (ANA), and albumin. On the other hand, whether immunosuppressants can improve the poor prognosis of MDA5^+^ DM-ILD is an important direction for future research. The recently proposed treat-to-target strategies in the latest juvenile dermatomyositis (JDM) guidelines, specifically the optimization of immunomodulatory therapy, may provide valuable insights for improving outcomes in MDA5^+^ DM-ILD (41). Future research should explore the application of similar strategies in adult DM-ILD populations. Although this meta-analysis specifically focused on MDA5^+^ DM-ILD patients, it is noteworthy that a recently reported case described an anti-MDA5 antibody-negative JDM patient who developed fatal RP-ILD within 1.5 months of diagnosis (42). This suggests that certain risk factors, particularly RP-ILD, may transcend serological status and warrant vigilance regardless of MDA5 antibody positivity.

There are some limitations in our pooled study. Firstly, among the included studies, the confounders adjusted for in the multivariable Cox models were inconsistent. Pooling these adjusted HRs could compromise the comparability and validity of the pooled analysis and potentially affect the reliability of the results. To avoid bias caused by differences in adjustment models, this study ultimately opted to pool risk factors derived from univariate Cox proportional hazards regression analyses. However, using only univariate HRs may influence the study findings, potentially leading to overestimation or underestimation of effects due to uncontrolled confounding. Future studies may consider conducting a meta-analysis based on multivariable Cox models derived from a sufficient number of included studies that have adjusted for the same set of potential confounding factors in order to guide patient management. Secondly, after a careful review of the 15 included studies, 11 explicit reports used all-cause mortality as the endpoint of the studies. The remaining four studies reported mortality rates related to respiratory failure. Given that respiratory failure-related deaths are a component of all-cause deaths, in this meta-analysis, all mortality rates reported in studies (including disease-specific mortality rates) were regarded as all-cause mortality rates. This method is statistically reasonable, but it may introduce potential heterogeneity. If there are significant non-respiratory failure deaths, it may lead to a slight underestimation of the true all-cause mortality rate. Thirdly, for the pooled estimates of some risk factors (for example, WBC, KL-6, ferritin (≥800 ng/mL), and lymphocyte count (<1.1×10^9^/L)), only two studies were concluded for each factor. The limited number of included studies may affect the stability and reliability of the aggregated results. Future large-scale studies are necessary to verify the reliability of these findings. Fourthly, there is significant heterogeneity in PaO_2_, FVC%, and RP-ILD, possibly due to differences in the severity of lung involvement, treatment strategies, and comorbidities. Since there are only three studies for each factor, conducting subgroup analysis may yield unreliable results. Therefore, we are unable to conduct a reliable and in-depth exploration of the sources of heterogeneity. Future studies could consider conducting subgroup analyses/meta-regressions on risk factors with significant heterogeneity (such as PaO_2_, FVC%, and RP-ILD) based on a sufficient number of included studies to further explore the sources of heterogeneity. In addition, after NOS literature quality evaluation, the studies included in this research demonstrated high quality, significantly enhancing the robustness of the results. However, future studies should place greater emphasis on controlling for key confounding factors (e.g., age) during design to enhance comparability, and improve follow-up rates/report response rates to reduce selection bias. Finally, although MDA5^+^ DM-ILD has been reported in different ethnic populations, it is mainly among the Japanese and the Chinese (5). Similarly, among the 15 studies included in our meta-analysis, 14 were from East Asia (China/Japan), which to some extent reflects that these regions have a higher prevalence rate. Therefore, there may be selection bias in our research. Future studies may consider conducting a meta-analysis in non-Asian populations to verify the general applicability of the pooled estimates in our research.

## Conclusion

In summary, the results of our pooled analysis demonstrated that age, smoking, fever, CRP, RP-ILD, WBC, KL-6, ferritin (≥800 ng/mL), and lymphocyte count (<1.1×10^9^/L) were associated with mortality in MDA5^+^ DM-ILD. Early intervention and management of risk factors associated with MDA5^+^ DM-ILD are crucial for improving the poor prognosis of patients.

## Data Availability

The original contributions presented in the study are included in the article/**Supplementary Material**. Further inquiries can be directed to the corresponding author.
