# Supplementary material for: Risk factors for mortality in anti-MDA5 antibody-positive dermatomyositis with interstitial lung disease: a systematic review and meta-analysis
Source: Front Immunol. 2025 Jul 17;16:1628748. doi: 10.3389/fimmu.2025.1628748 (PMC12310698; doi:10.3389/fimmu.2025.1628748)
Supplement: Supplementary file 1 [file Table1.docx]

Supplementary Table 1. Database and Search strategy

| **Database** | **Search strategy** |
| --- | --- |
| **Pubmed**  **(n=691)** | #1:((((((((((((Dermatomyositis[MeSH Terms]) OR (Dermatomyositis[Title/Abstract])) OR (Dermatomyositis[Title/Abstract])) OR (Polymyositis Dermatomyositis[Title/Abstract])) OR (Dermatopolymyositis[Title/Abstract])) OR (Dermatomyositis, Adult Type[Title/Abstract])) OR (Adult Type Dermatomyositis[Title/Abstract])) OR (Dermatomyositis, Childhood Type[Title/Abstract])) OR (Childhood Type Dermatomyositis[Title/Abstract])) OR (Juvenile Dermatomyositis[Title/Abstract])) OR (Dermatomyositis, Juvenile[Title/Abstract])) OR (Juvenile Myositis[Title/Abstract])) OR (Myositis, Juvenile[Title/Abstract])  =13541 items |
|  | #2:((MDA5[Title/Abstract]) OR (MDA-5[Title/Abstract])) OR (melanoma differentiation-associated gene-5[Title/Abstract])  =3016 items |
|  | #3:(((((((((((((Lung Diseases, Interstitial[MeSH Terms]) OR (Diffuse Parenchymal Lung Disease[Title/Abstract])) OR (Interstitial Lung Disease[Title/Abstract])) OR (Lung Disease, Interstitial[Title/Abstract])) OR (Pneumonia, Interstitial[Title/Abstract])) OR (Interstitial Pneumonia[Title/Abstract])) OR (Pneumonias, Interstitial[Title/Abstract])) OR (Pneumonitis, Interstitial[Title/Abstract])) OR (Interstitial Pneumonitis[Title/Abstract])) OR (Pneumonitides, Interstitial[Title/Abstract]))) )) OR (ILD[Title/Abstract])  =103662 items |
|  | #4:#1 AND #2 AND #3  =691 items |
| **Embase**  **(n=1329)** | #1:'dermatomyositis'/exp OR 'dermato-myositis':ab,ti OR 'dermatomucomyositis':ab,ti OR 'dermatomyositides':ab,ti OR 'dermatopolymyositis':ab,ti OR 'polymyositis arthropathica':ab,ti OR 'Unverricht-Wagner syndrome':ab,ti OR 'Wagner-Unverricht syndrome':ab,ti OR 'Wegner Hepp Unverrricht disease':ab,ti OR 'dermatomyositis':ab,ti  =24142 items |
|  | #2: 'mda5'/exp OR 'mda-5':ab,ti OR 'melanoma differentiation-associated gene-5':ab,ti  =4612 items |
|  | #3:'interstitial lung disease'/exp OR 'lung diseases, interstitial':ab,ti OR 'diffuse parenchymal lung disease':ab,ti OR 'interstitial lung disease':ab,ti OR 'interstitial pneumonia':ab,ti OR 'ild':ab,ti  =161556 items |
|  | #4:#1 AND #2 AND #3  =1329 items |
| **Web of science**  **(n=1077)** | #1: TS= (Dermatomyositis OR Dermatomyositis OR Dermatomyositis OR Polymyositis Dermatomyositis OR Dermatopolymyositis OR Dermatomyositis, Adult Type OR Adult Type Dermatomyositis OR Dermatomyositis, Childhood Type OR Childhood Type Dermatomyositis OR Juvenile Dermatomyositis OR Dermatomyositis, Juvenile OR Juvenile Myositis OR Myositis, Juvenile)  =20769 items |
|  | #2: TS= (MDA5 OR MDA-5 OR melanoma differentiation-associated gene-5)  =4757 items |
|  | #3: TS= (Lung Diseases, Interstitial OR Diffuse Parenchymal Lung Disease OR Interstitial Lung Disease OR Interstitial Pneumonia OR ILD) =63719 items |
|  | #4:#1 AND #2 AND #3  =1077 items |
| **Scopus**  **(n=848)** | #1: TITLE-ABS-KEY ("Dermatomyositis" OR "Dermatomyositis" OR "Dermatomyositis" OR "Polymyositis Dermatomyositis" OR "Dermatopolymyositis" OR "Dermatomyositis, Adult Type" OR "Adult Type Dermatomyositis" OR "Dermatomyositis, Childhood Type" OR "Childhood Type Dermatomyositis" OR "Juvenile Dermatomyositis" OR "Dermatomyositis, Juvenile" OR "Juvenile Myositis" OR "Myositis, Juvenile")  =20181 items |
|  | #2: TITLE-ABS-KEY ( "MDA5" OR "MDA-5" OR "melanoma differentiation-associated gene-5")  =3290 items |
|  | #3: TITLE-ABS-KEY ("lung diseases, interstitial" OR "diffuse parenchymal lung disease" OR "interstitial lung disease" OR "interstitial pneumonia" OR "ild")  =54269 items |
|  | #4:#1 AND #2 AND #3  =848 items |

Supplementary Table 2 Definitions of Rapidly Progressive Interstitial Lung Disease (RP-ILD) in Included Studies

| Study | Definition |
| --- | --- |
| Zhou W, et al. 2024 | RP-ILD was diagnosed when the following conditions were met within the past 4 weeks: a rapidly deterioration of respiratory symptoms leading to severe hypoxic respiratory failure with arterial partial pressure of oxygen to fraction of inspired oxygen (PaO_2_/FiO_2_) < 300mmHg; newly-emerging abnormalities on chest HRCT including the ground-glass opacities or consolidations or the reticulation or honeycombing with the exclusion of identified causes, such as acute heart failure or pulmonary atrial embolism, etc. (1). |
| Lian X, et al. 2023 | RPILD was characterized by progressive dyspnoea and hypoxaemia, with a worsening of radiologic changes of interstitial lung inflammation within 3 months after the onset of respiratory symptoms (2). |
| Liu H, et al. 2024 | RPILD was defined as a progressive deterioration of interstitial changes within 3 months based on the radiological assessment of HRCT examination (3). |

Supplementary Table 3 Results of Newcastle-Ottawa quality assessment Scale for each included study

| **Criteria** | | | **Mao M, et al. 2020** | **Li Y, et al. 2023** | **Xu W, et al. 2021** | **Yamaguchi K, et al. 2022** | **Fukada A, et al. 2024** | **Zhou W, et al. 2024** | **Li Y, et al. 2020** | **Lian X, et al. 2023** | **Waseda Y, et al. 2022** | **Liu H, et al. 2024** | **Gui X, et al. 2021** | **Bay P, et al. 2022** | **Ye Y, et al. 2019** | **Fujisawa T, et al. 2019** | **Zhao S, et al. 2022** |
| --- | --- | --- | --- | --- | --- | --- | --- | --- | --- | --- | --- | --- | --- | --- | --- | --- | --- |
| **Selection** | 1. Clear definition of the disease. | | ★ | ★ | ★ | ★ | ★ | ★ | ★ | ★ | ★ | ★ | ★ | ★ | ★ | ★ | ★ |
|  | 2. The representativeness of the selected cases. | | ★ | ★ | ★ | ★ | ★ | ★ | ★ | ★ | ★ | ★ | ★ | ★ | ★ | ★ | ★ |
|  | 3. Selection of the control group. | | ★ | ★ | ★ | ★ | ★ | ★ | ★ | ★ | ★ | ★ | ★ | ★ | ★ | ★ | ★ |
|  | 4. The definition of the control group. | | ★ | ★ | ★ | ★ | ★ | ★ | ★ | ★ | ★ | ★ | ★ | ★ | ★ | ★ | ★ |
| **Comparability** | 5. The comparability of cases and controls obtained based on design or analysis. | Important factors (e.g. age) |  |  |  |  |  | ★ | ★ | ★ |  | ★ | ★ |  |  | ★ | ★ |
|  |  | Confounding factors | ★ | ★ | ★ | ★ | ★ | ★ | ★ | ★ | ★ | ★ | ★ | ★ | ★ | ★ | ★ |
| **Outcome** | 6. Identification of exposure factors. | | ★ | ★ | ★ | ★ | ★ | ★ | ★ | ★ | ★ | ★ | ★ | ★ | ★ | ★ | ★ |
|  | 7. The same method was used to determine the exposure and control factors. | | ★ | ★ | ★ | ★ | ★ | ★ | ★ | ★ | ★ | ★ | ★ | ★ | ★ | ★ | ★ |
|  | 1. Non-response rate. | |  |  | ★ |  |  |  | ★ | ★ | ★ |  |  |  |  |  |  |
| **Total** | | | 7★ | 7★ | 8★ | 7★ | 7★ | 8★ | 9★ | 9★ | 8★ | 8★ | 8★ | 7★ | 7★ | 8★ | 8★ |

Supplementary Figure 1 Plot for the assessment of heterogeneity among the included studies of age for mortality in MDA5^+^ DM-ILD through One-by-one elimination method


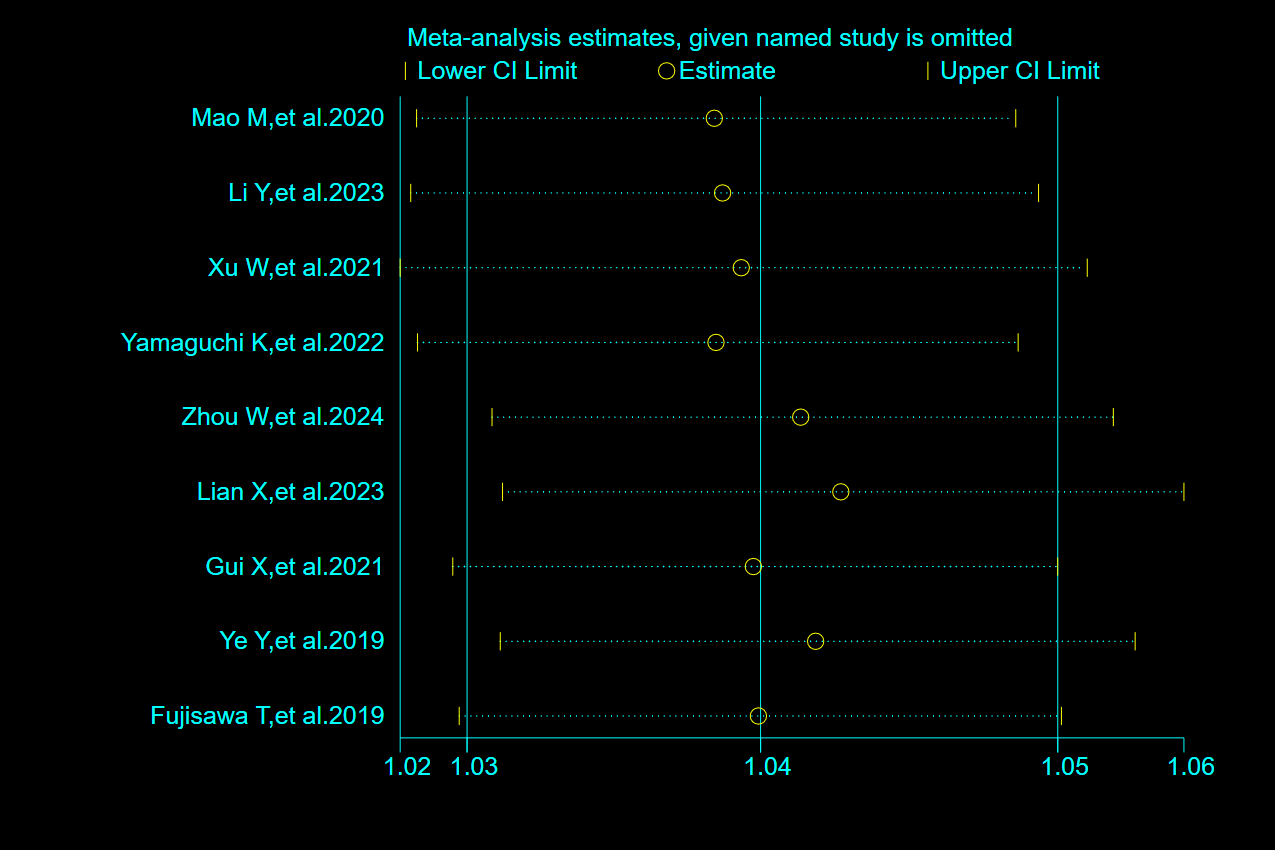


Supplementary Figure 2 Plot for the assessment of heterogeneity among the included studies of male for mortality in MDA5^+^ DM-ILD through One-by-one elimination method


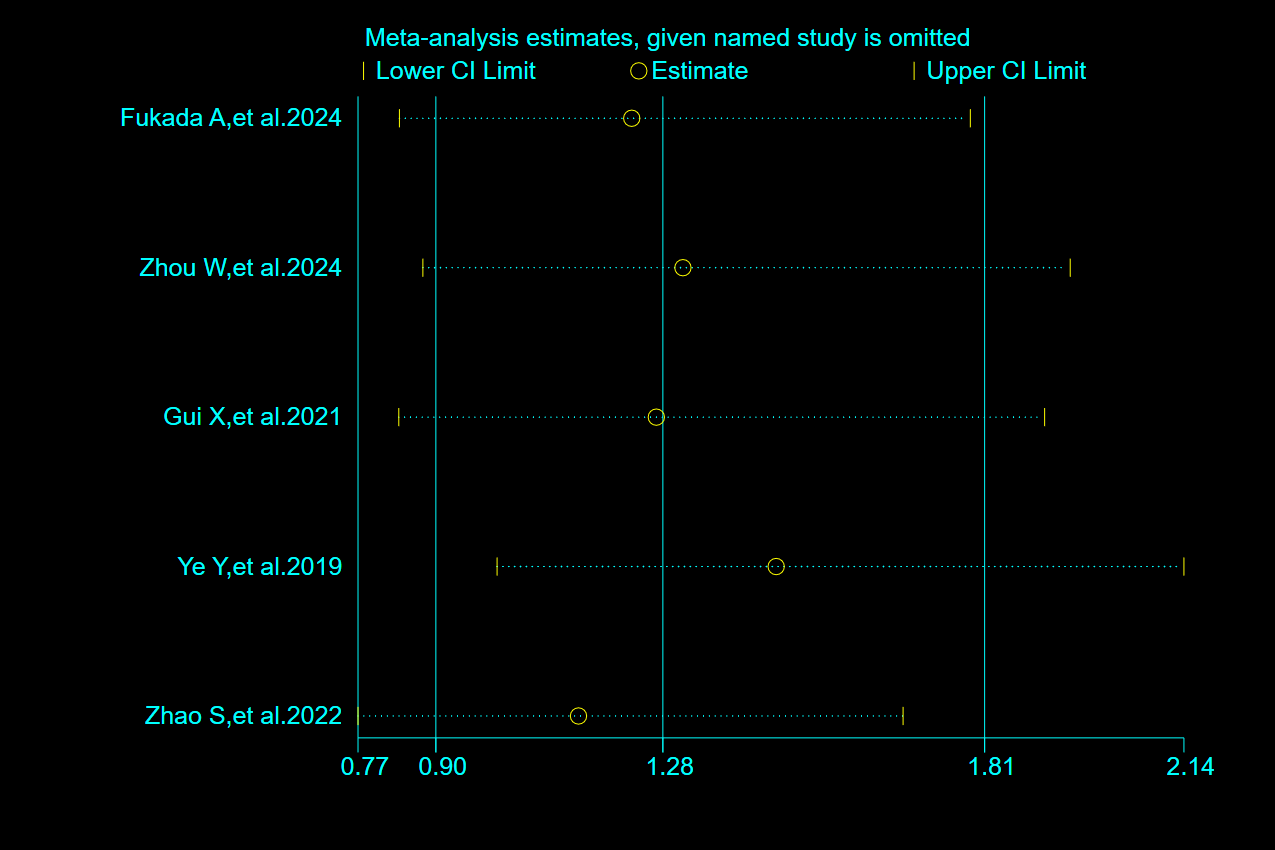


Supplementary Figure 3 Plot for the assessment of heterogeneity among the included studies of smkoing for mortality in MDA5^+^ DM-ILD through One-by-one elimination method


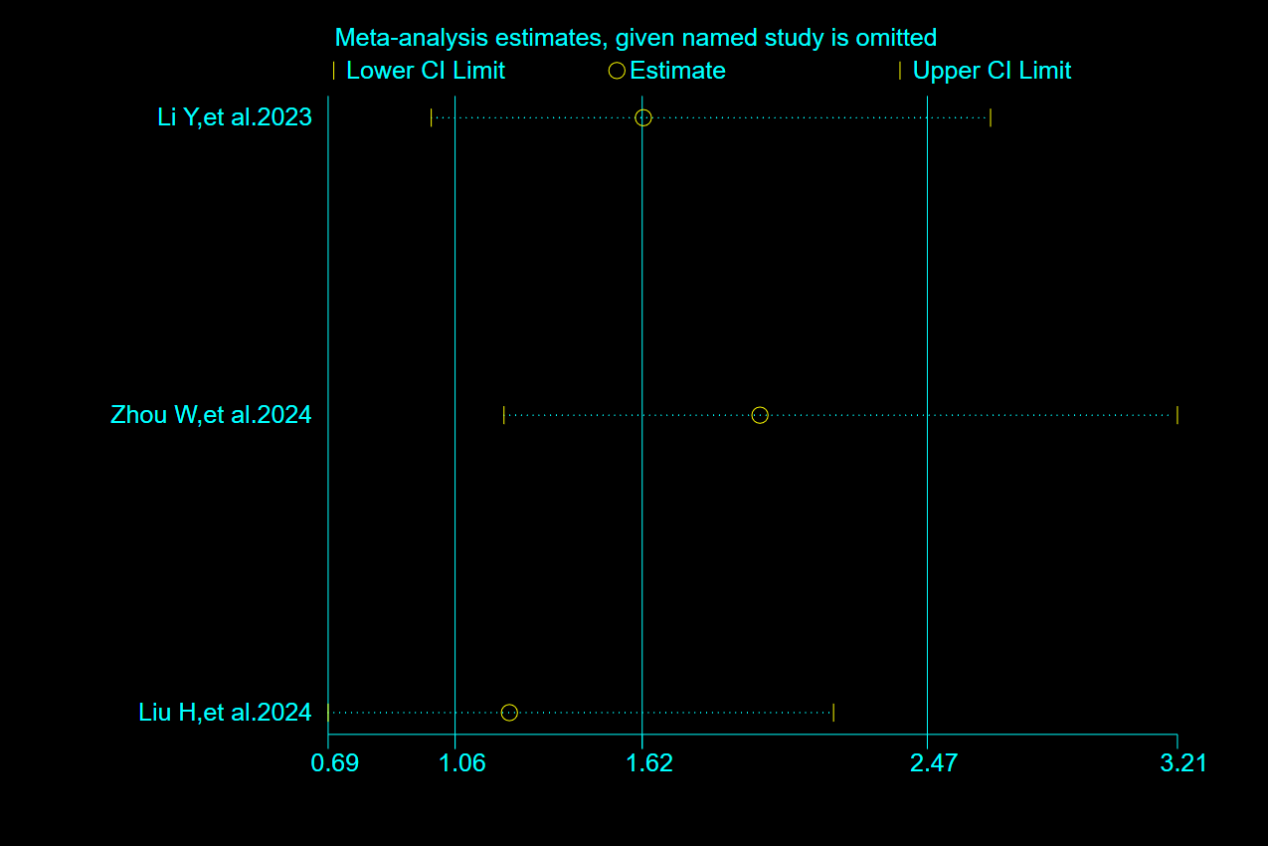


Supplementary Figure 4 Plot for the assessment of heterogeneity among the included studies of fever for mortality in MDA5^+^ DM-ILD through One-by-one elimination method


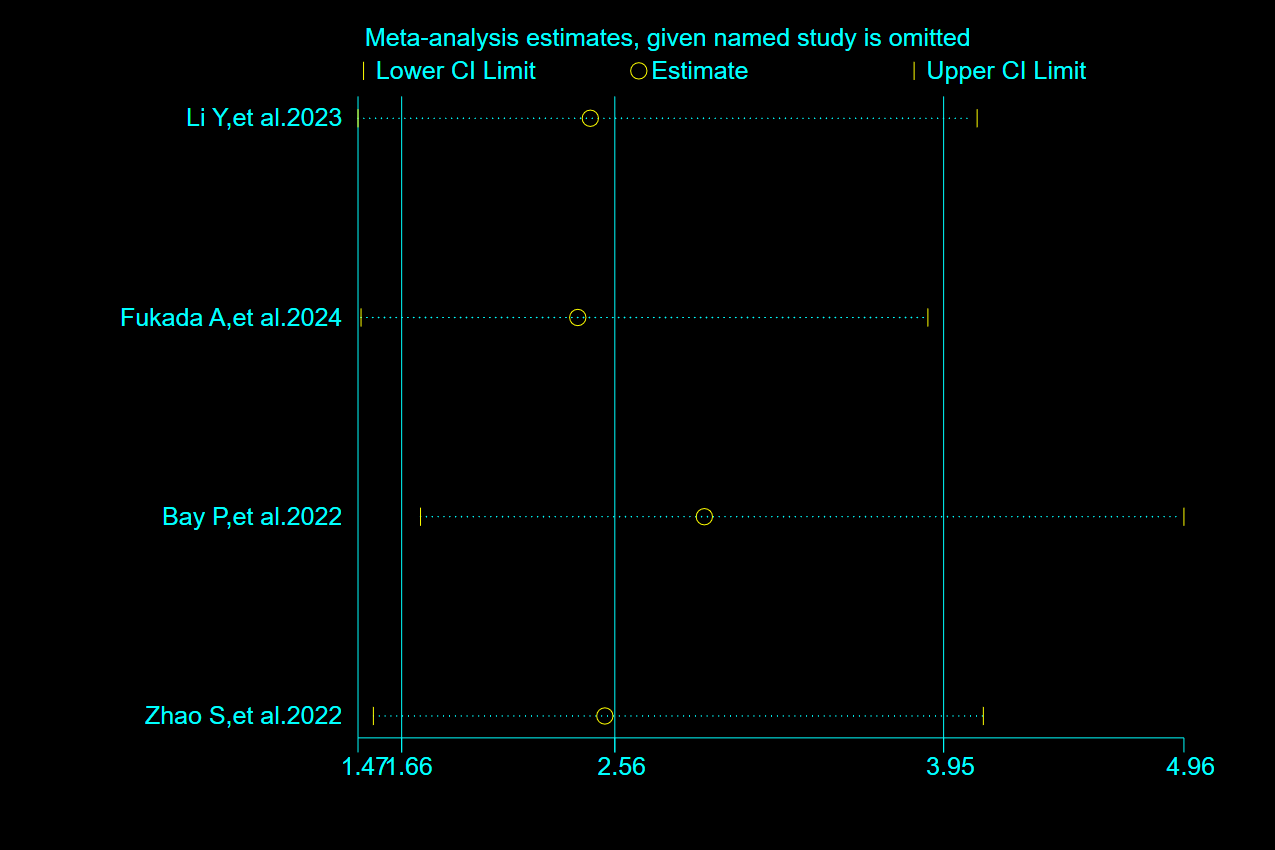


Supplementary Figure 5 Plot for the assessment of heterogeneity among the included studies of CRP for mortality in MDA5^+^ DM-ILD through One-by-one elimination method


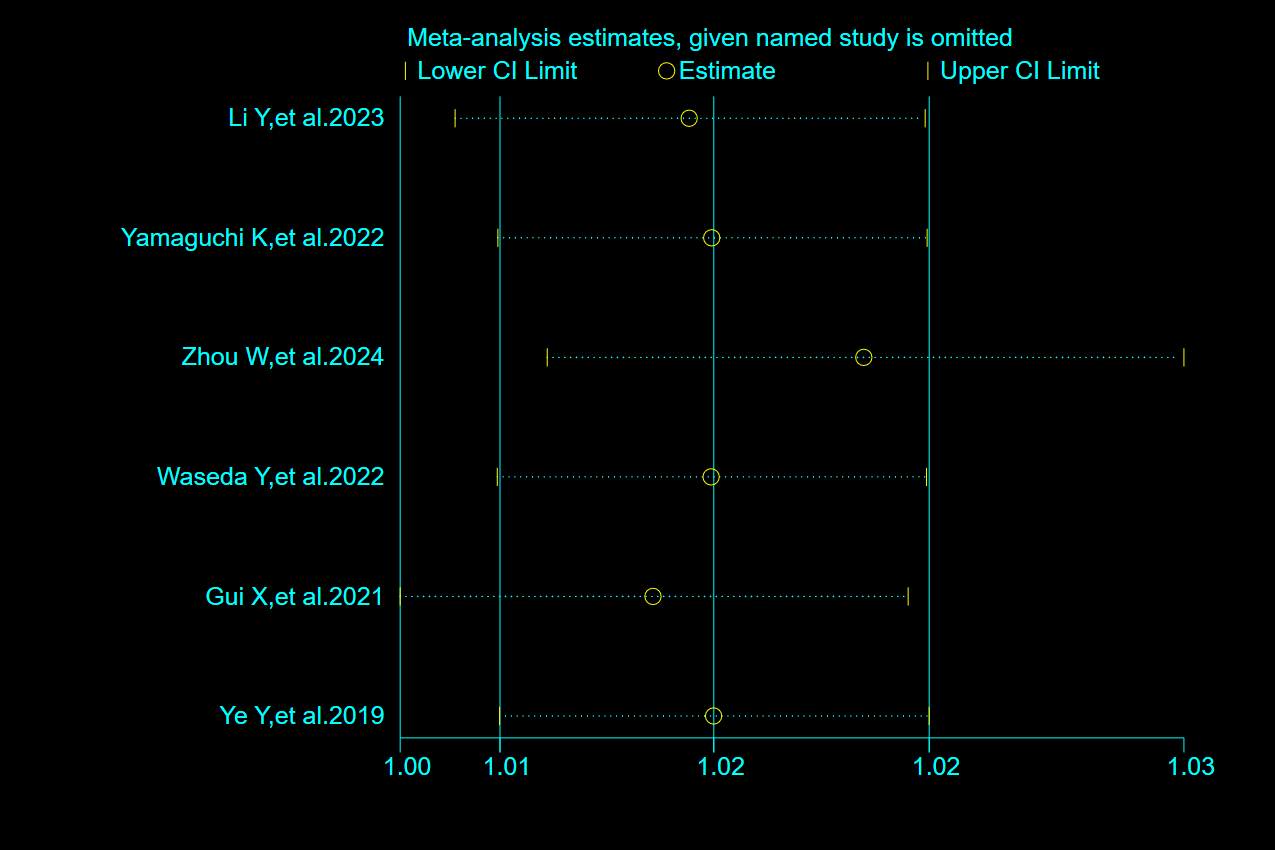


Supplementary Figure 6 Plot for the assessment of heterogeneity among the included studies of CK for mortality in MDA5^+^ DM-ILD through One-by-one elimination method


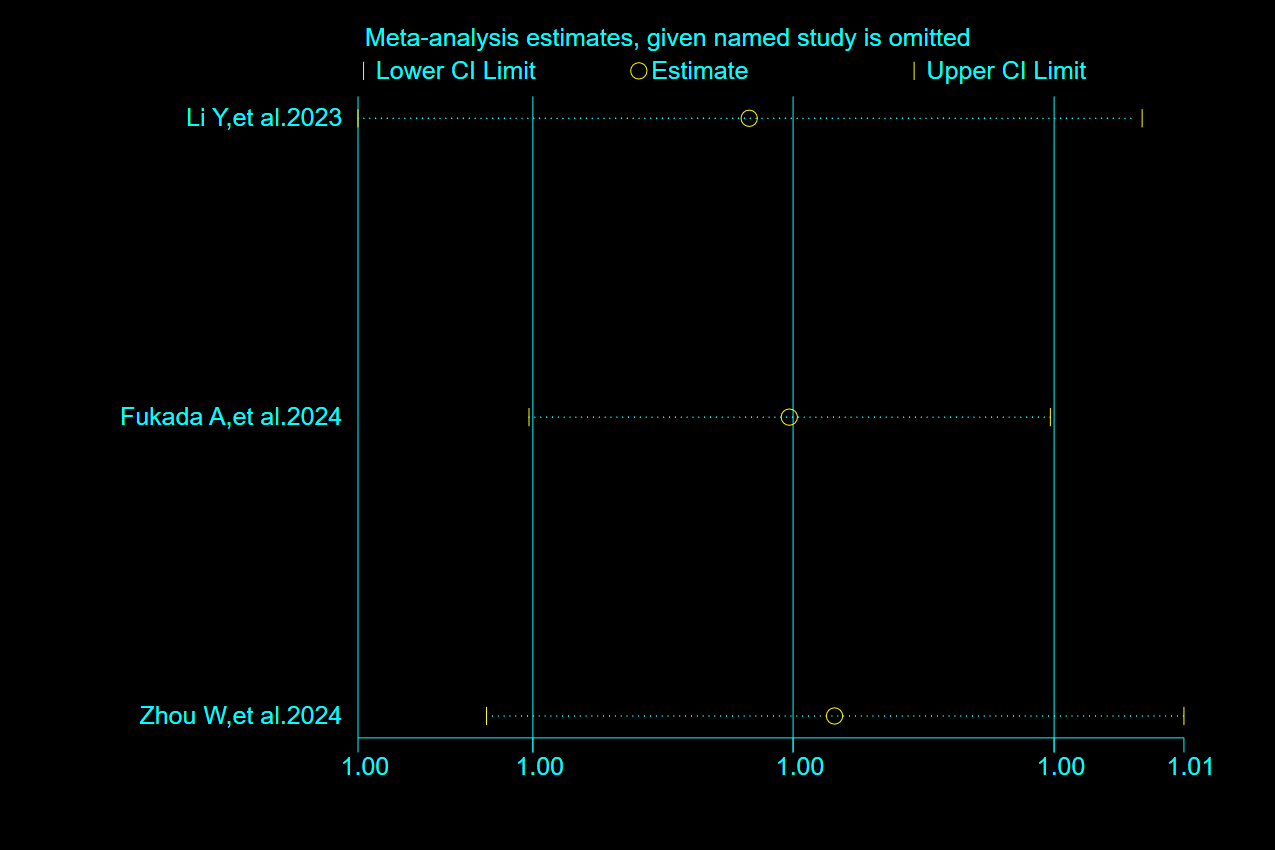


Supplementary Figure 7 Plot for the assessment of heterogeneity among the included studies of DLCO% for mortality in MDA5^+^ DM-ILD through One-by-one elimination method


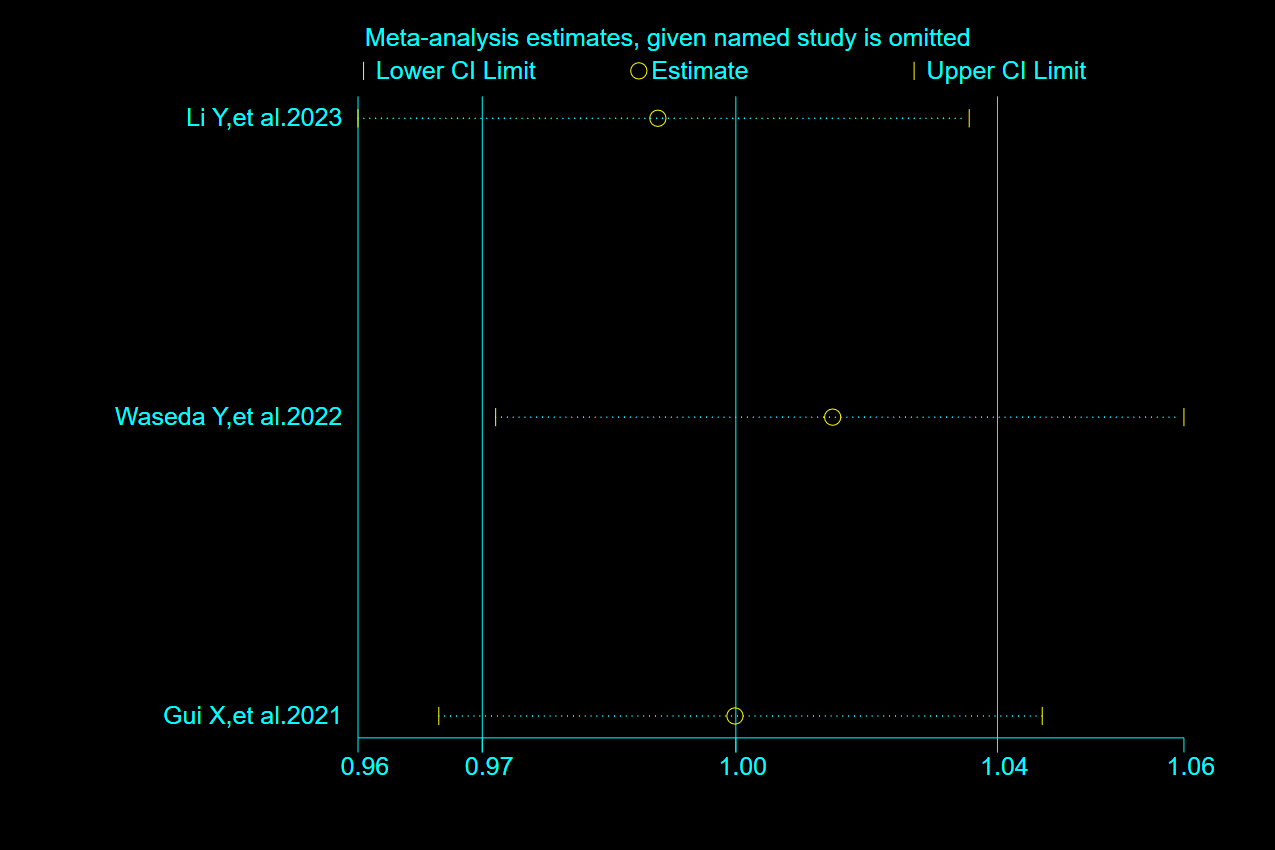


Supplementary Figure 8 Plot for the assessment of heterogeneity among the included studies of PaO_2_ for mortality in MDA5^+^ DM-ILD through One-by-one elimination method


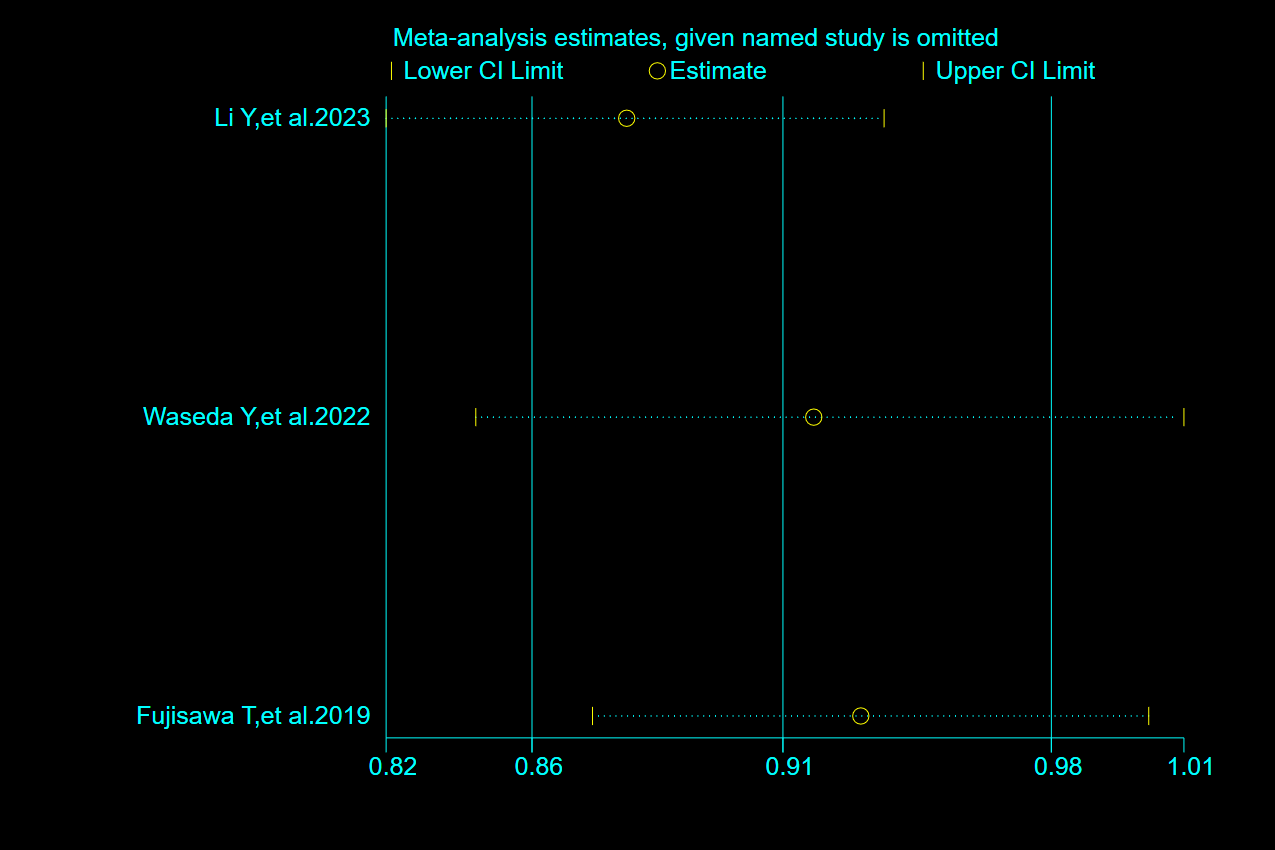


Supplementary Figure 9 Plot for the assessment of heterogeneity among the included studies of FVC% for mortality in MDA5^+^ DM-ILD through One-by-one elimination method


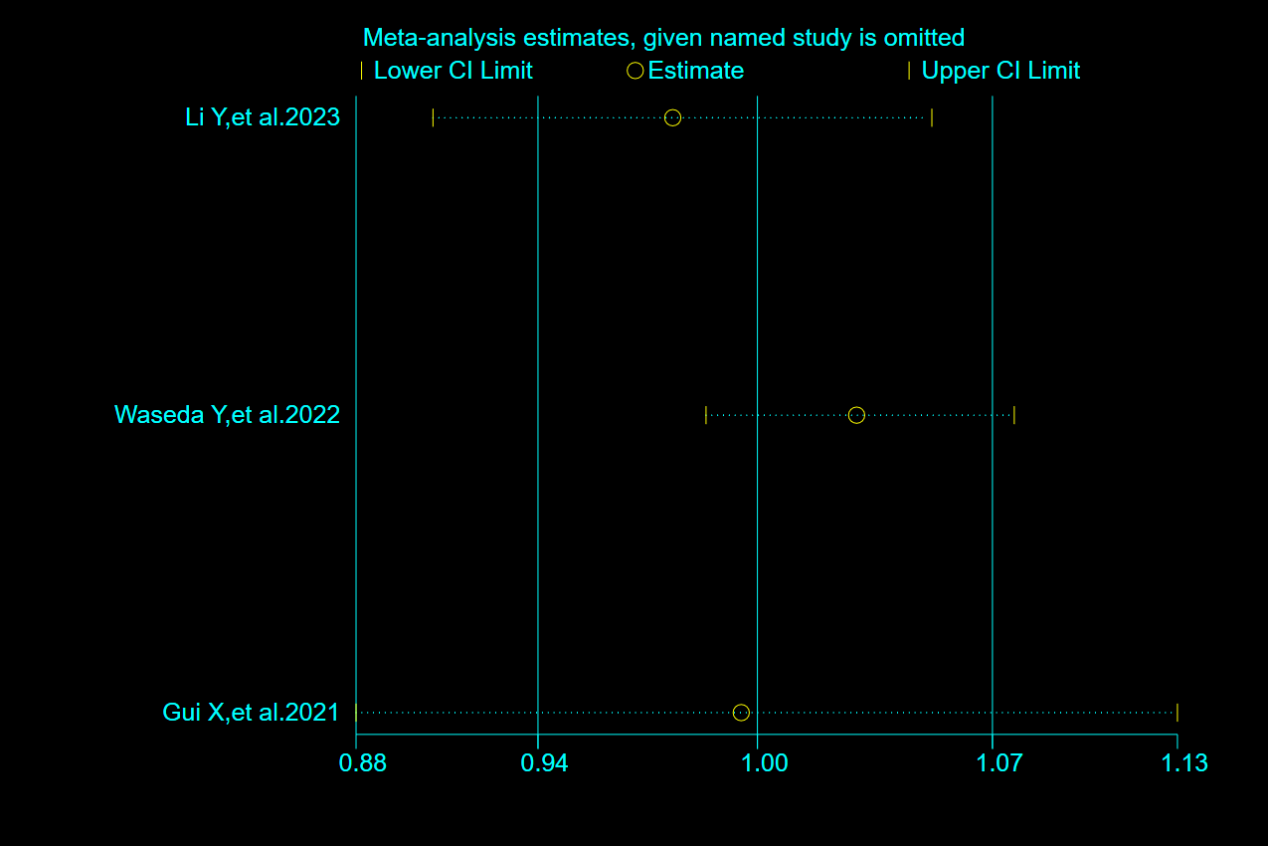


Supplementary Figure 10 Plot for the assessment of heterogeneity among the included studies of RP-ILD for mortality in MDA5^+^ DM-ILD through One-by-one elimination method


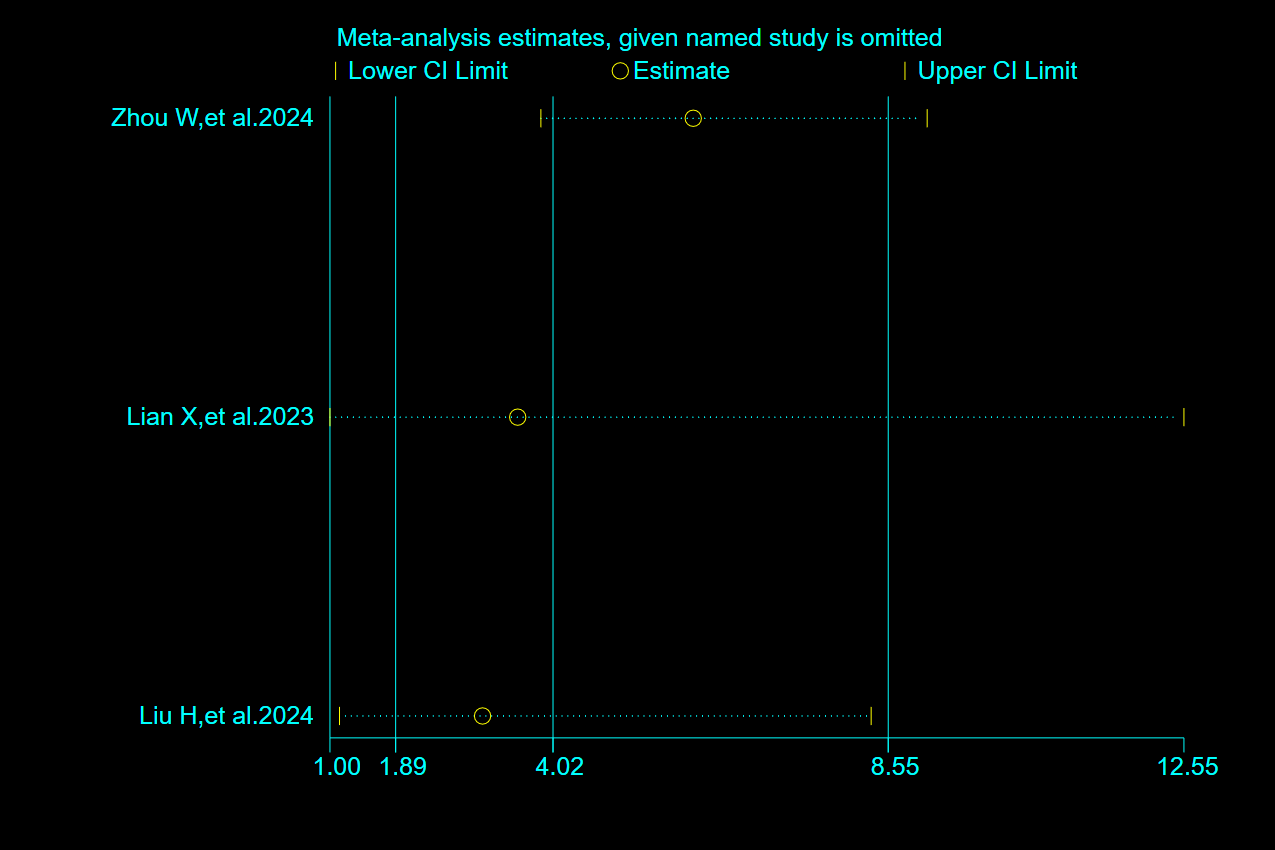


Supplementary Figure 11 Plot for the assessment of heterogeneity among the included studies of ESR for mortality in MDA5^+^ DM-ILD through One-by-one elimination method


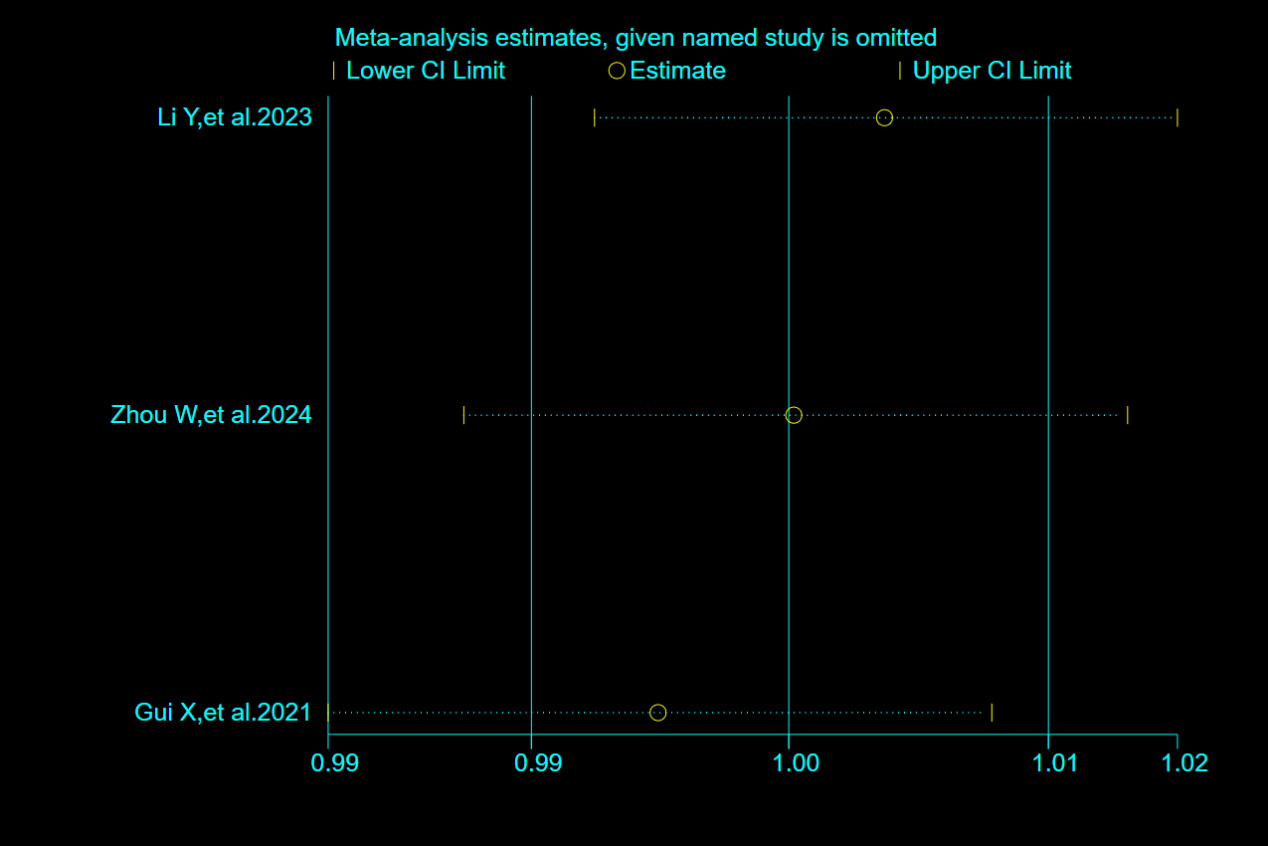


Supplementary Figure 12 Plot for the assessment of heterogeneity among the included studies of WBC for mortality in MDA5^+^ DM-ILD through One-by-one elimination method


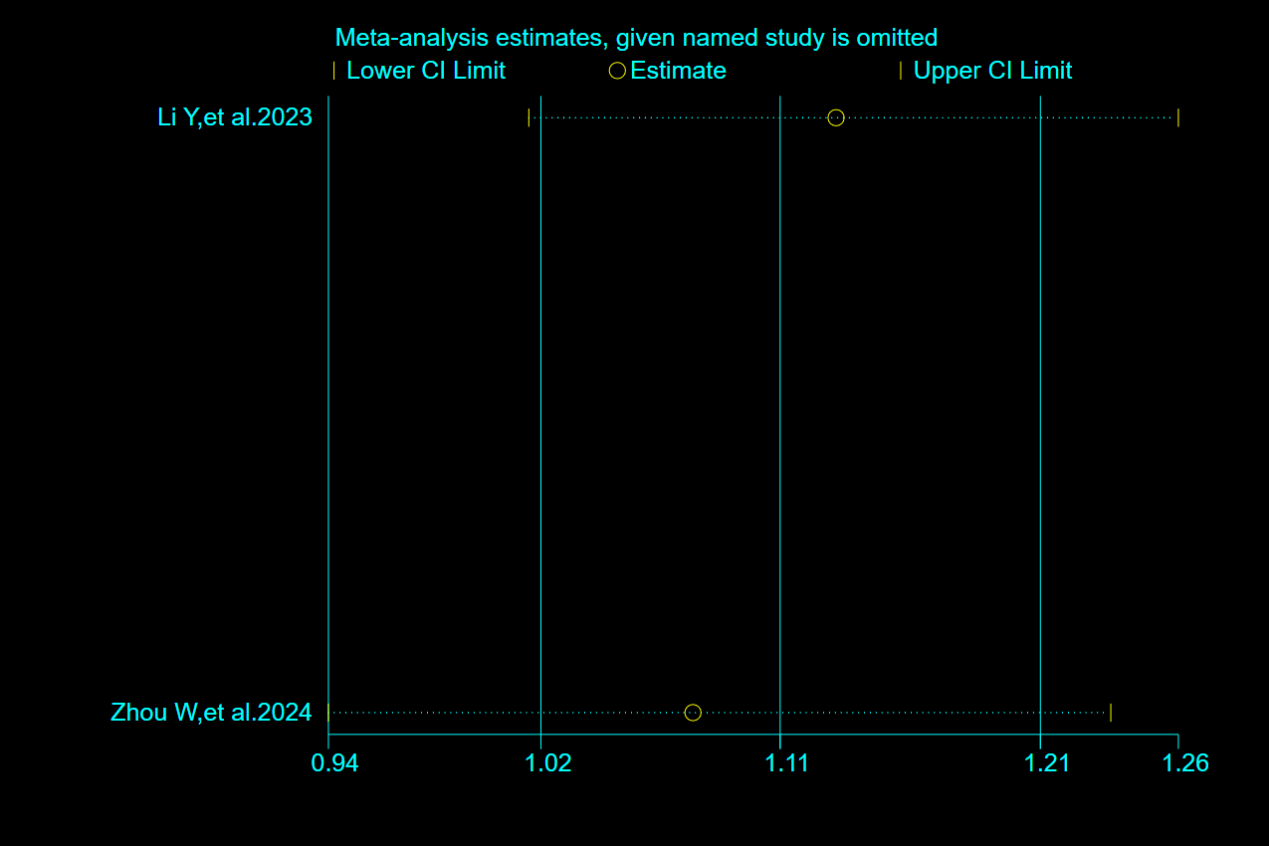


Supplementary Figure 13 Plot for the assessment of heterogeneity among the included studies of KL-6 for mortality in MDA5^+^ DM-ILD through One-by-one elimination method


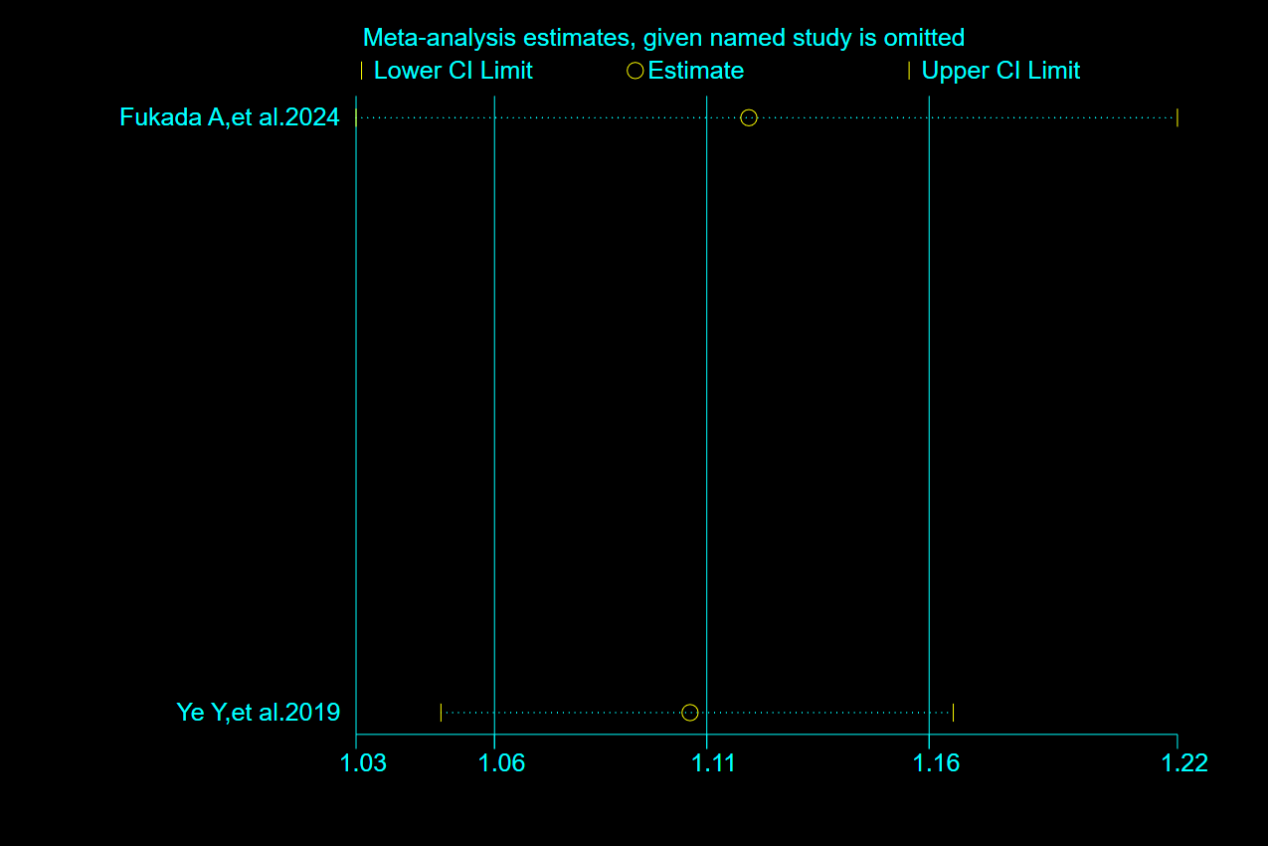


Supplementary Figure 14 Plot for the assessment of heterogeneity among the included studies of ferritin ≥ 800 ng/mL for mortality in MDA5^+^ DM-ILD through One-by-one elimination method


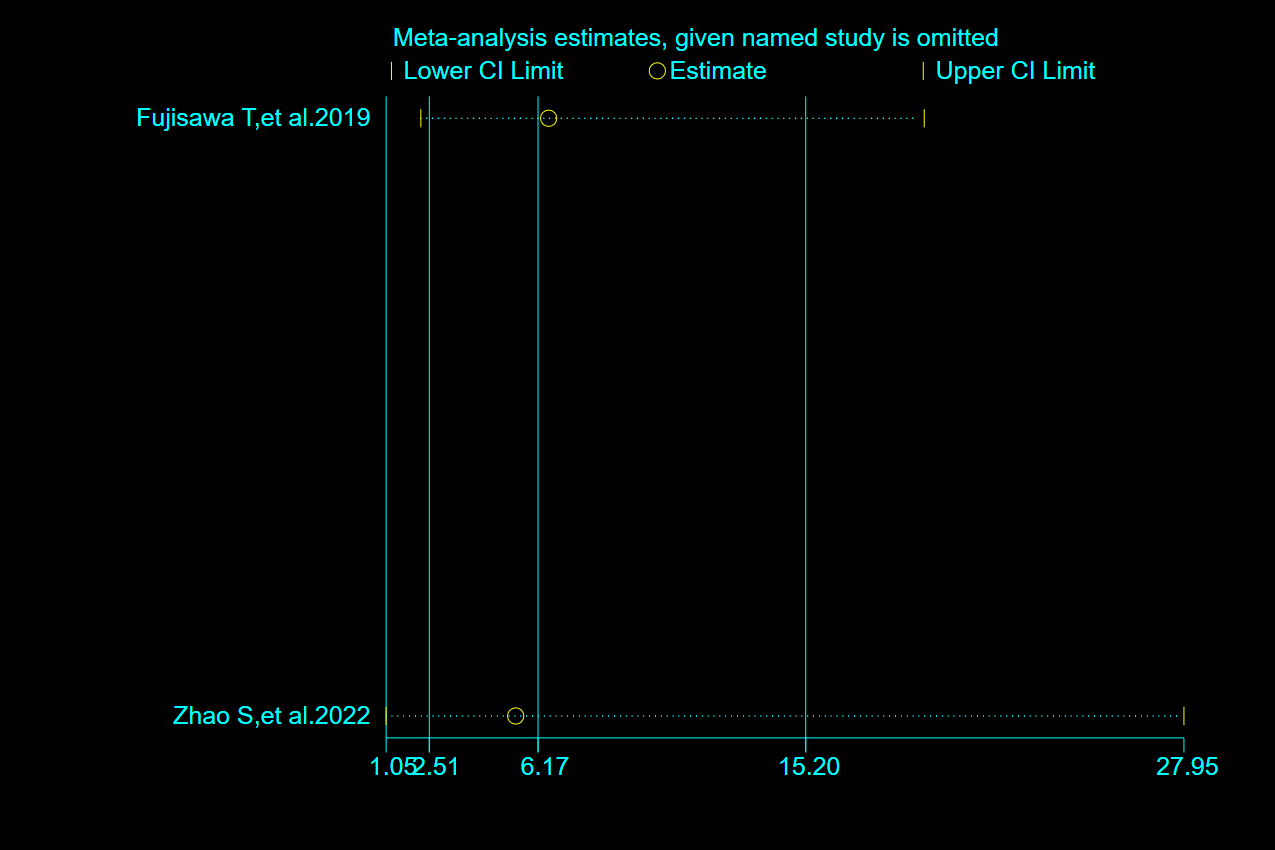


Supplementary Figure 15 Plot for the assessment of heterogeneity among the included studies of lymphocyte count<1.1×10^9^/L for mortality in MDA5^+^ DM-ILD through One-by-one elimination method


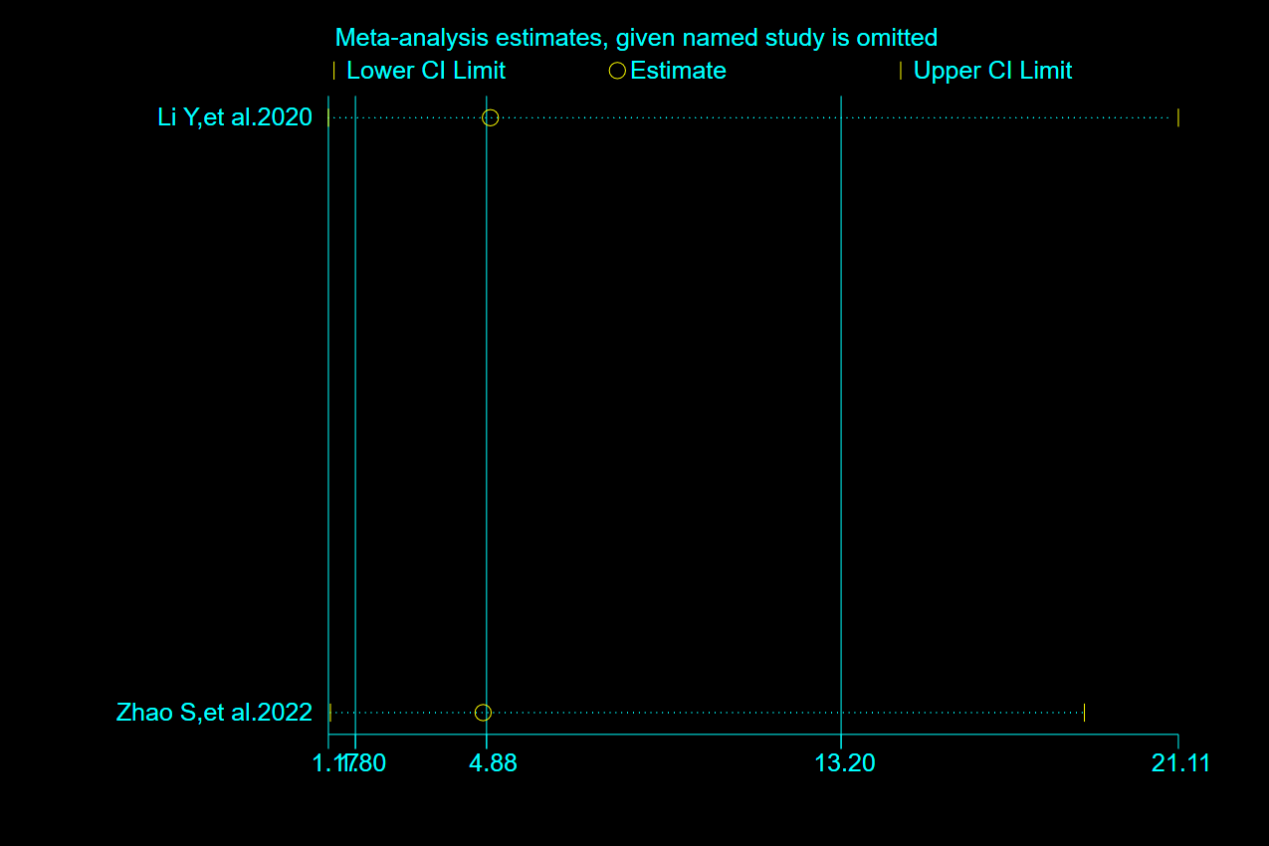


Supplementary Figure 16 Funnel plots of trim-and-fill analysis for hazard ratio predicting mortality of MDA5^+^ DM-ILD (A: male, B: smoking, C: PaO_2_, D: FVC%, E: RP-ILD, F: ESR, G: WBC, H: KL-6, I: ferritin (≥ 800 ng/mL), J: lymphocyte count (< 1.1×10⁹/L)).


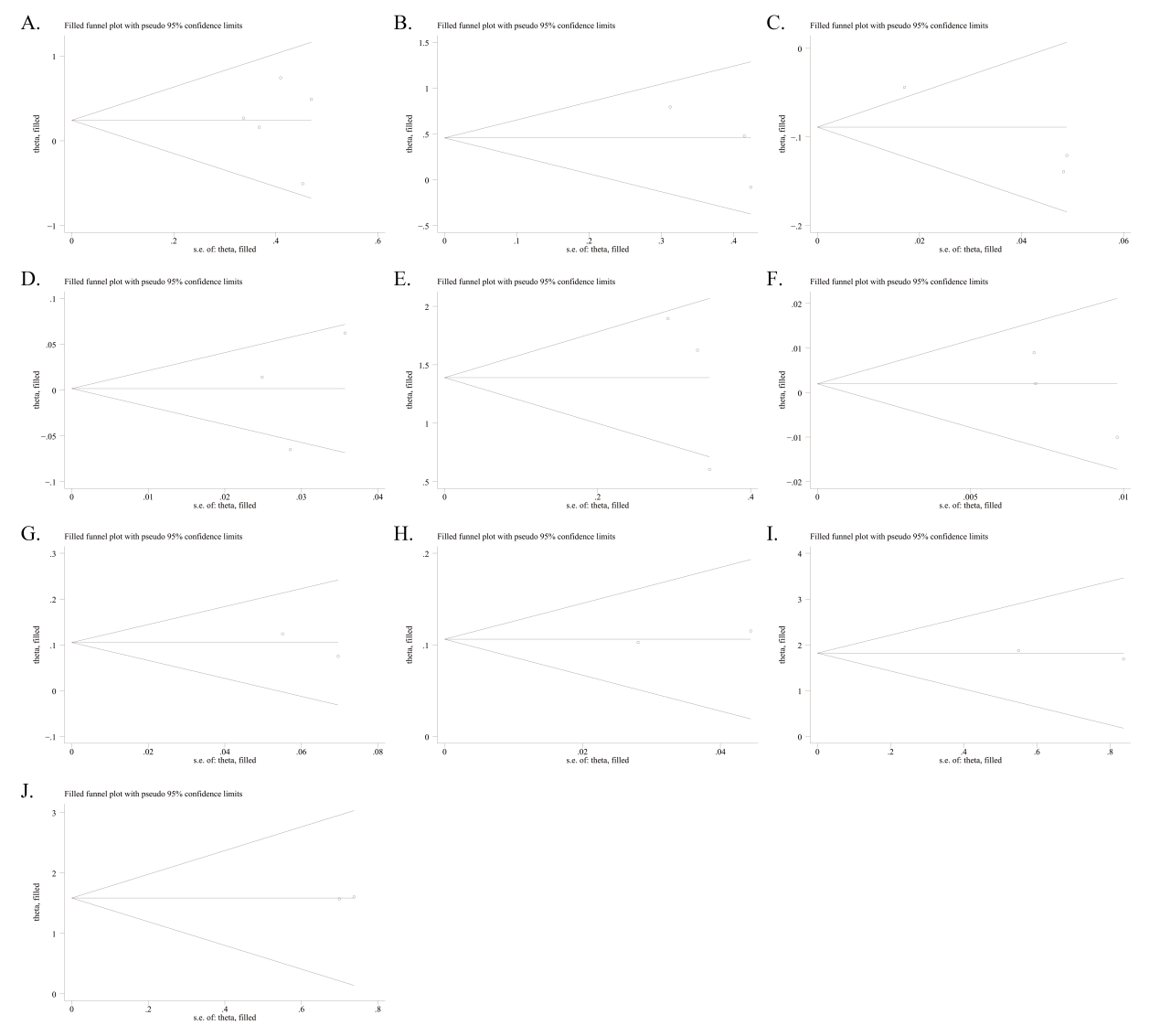


MDA5: melanoma differentiation-associated gene 5; DM: dermatomyositis; ILD: interstitial lung disease; FVC%: percent predicted forced vital capacity; RP-ILD: rapidly-progressive interstitial lung disease; ESR: erythrocyte sedimentation rate; WBC: white blood cell count; KL-6: Krebs von den Lungen-6.

**References**

1. Chen Z, Cao M, Plana MN, Liang J, Cai H, Kuwana M, et al. Utility of anti-melanoma differentiation-associated gene 5 antibody measurement in identifying patients with dermatomyositis and a high risk for developing rapidly progressive interstitial lung disease: a review of the literature and a meta-analysis. *Arthritis Care Res (Hoboken)* (2013) **65**: 1316-24. doi:10.1002/acr.21985

2. Won HJ, Soon KD, Keun LC, Yoo B, Bum SJ, Kitaichi M, et al. Two distinct clinical types of interstitial lung disease associated with polymyositis-dermatomyositis. *Respir Med* (2007) **101**: 1761-69. doi:10.1016/j.rmed.2007.02.017

3. Ichikado K, Suga M, Müller NL, Taniguchi H, Kondoh Y, Akira M, et al. Acute interstitial pneumonia: comparison of high-resolution computed tomography findings between survivors and nonsurvivors. *Am J Respir Crit Care Med* (2002) **165**: 1551-56. doi:10.1164/rccm.2106157
